# MmuPV1 E6 induces cell proliferation and other hallmarks of cancer

**DOI:** 10.1128/mbio.02458-23

**Published:** 2023-10-31

**Authors:** James C. Romero-Masters, Laura K. Muehlbauer, Mitchell Hayes, Miranda Grace, Evgenia Shishkova, Joshua J. Coon, Karl Munger, Paul F. Lambert

**Affiliations:** 1 McArdle Laboratory for Cancer Research, University of Wisconsin School of Medicine and Public Health, Madison, Wisconsin, USA; 2 Departments of Chemistry and Biomolecular Chemistry, University of Wisconsin-Madison, Madison, Wisconsin, USA; 3 Department of Developmental, Molecular and Chemical Biology, Tufts University School of Medicine, Boston, Massachusetts, USA; 4 Morgridge Institute for Research, Madison, Wisconsin, USA; Johns Hopkins University School of Medicine, Baltimore, Maryland, USA

**Keywords:** papillomavirus, cell proliferation, E6

## Abstract

The E6 protein encoded by the murine papillomavirus (MmuPV1) is essential for MmuPV1-induced skin disease. Our previous work has identified a number of cellular interacting partners of MmuPV1 E6 and E7 through affinity purification/mass spectrometry analysis. These studies revealed that MmuPV1 E6 potently inhibits keratinocyte differentiation through multiple molecular mechanisms including inhibition of NOTCH and TGF-β signaling. Here, we report that MmuPV1 E6 has additional important oncogenic activities when expressed in its natural host cells, mouse keratinocytes, including increasing proliferation, overcoming density-mediated growth arrest, and proliferation under conditions of limited supply of growth factors. Unbiased proteomic/transcriptomic analyses of mouse keratinocytes expressing MmuPV1 E6 substantiated its effect on these cellular processes and divulged that some of these effects may be mediated in part through it upregulating E2F activity. Our analyses also revealed that MmuPV1 E6 may alter other cancer hallmarks including evasion of growth suppressors, inhibition of immune response, resistance to cell death, and alterations in DNA damage response. Collectively, our results suggest that MmuPV1 E6 is a major driver of multiple hallmarks of cancer in MmuPV1’s natural host cells, mouse keratinocytes.

**IMPORTANCE:**

The Mus musculus papillomavirus 1 (MmuPV1) E6 and E7 proteins are required for MmuPV1-induced disease. Our understanding of the activities of MmuPV1 E6 has been based on affinity purification/mass spectrometry studies where cellular interacting partners of MmuPV1 E6 were identified, and these studies revealed that MmuPV1 E6 can inhibit keratinocyte differentiation through multiple mechanisms. We report that MmuPV1 E6 encodes additional activities including the induction of proliferation, resistance to density-mediated growth arrest, and decreased dependence on exogenous growth factors. Proteomic and transcriptomic analyses provided evidence that MmuPV1 E6 increases the expression and steady state levels of a number of cellular proteins that promote cellular proliferation and other hallmarks of cancer. These results indicate that MmuPV1 E6 is a major driver of MmuPV1-induced pathogenesis.

## INTRODUCTION

Human papillomavirus (HPV) infections are major human carcinogens causing nearly 5% of all human cancers (1–4). HPV causes a number of different malignancies in various organs that contain stratified squamous epithelial tissues including cervical and other anogenital tract cancers, head and neck cancer, and non-melanoma skin cancer (2–9). HPVs can be categorized into mucosal and cutaneous HPVs based on which tissue types they predominately infect (5). Cutaneous HPVs cause benign hyperplastic lesions known as warts but a subset of cutaneous HPVs, predominately HPV 5 and 8, can cause non-melanoma skin cancer in individuals with the hereditary condition epidermodysplasia verruciformis (EV) or long-term immunosuppressed organ transplant patients (6, 8–13). In addition, a subset of mucosal HPVs referred to as high-risk HPVs cause lesions in mucosal tissues that if allowed to persist can progress to anogenital and head and neck cancer (1–4, 14). The low-risk mucosal HPVs cause benign disease specifically genital and oral warts.

The most well-studied HPV proteins are E5, E6, and E7 proteins which are critical for viral replication because they modulate the host cells to make them more amenable to viral replication, that is, promoting DNA synthesis, delaying differentiation, and inhibiting immune responses. The high-risk mucosal HPV E6 and E7 proteins target critical tumor suppressors specifically p53 and pRB, thereby dampening their tumor suppressive activities (15, 16, 16–18). High-risk HPV E7 proteins interact with pRB through an LXCXE motif and have been shown to promote pRB degradation (19, 20). The degradation of pRB by high-risk mucosal HPV E7 leads to the activation of the E2F transcription factors that promote the expression of genes critical for DNA replication and cell division. High-risk HPV E6 proteins target p53 for degradation by interacting with the E6AP ubiquitin ligase muting cellular responses to E7’s degradation of pRB, such as apoptosis or senescence, to ensure cell survival (18, 21, 22). While these activities of the high-risk HPV E6 and E7 proteins are critical for viral pathogenesis, they also target additional cellular proteins including PDZ proteins, UBR4, and PTPN14 that are critical for viral pathogenesis (23–31). Mucosal HPVs encode a third viral oncoprotein, E5, that promotes EGFR signaling as well as affects other cellular pathways (32, 33). The ability of high-risk HPV and some cutaneous HPVs to promote the development of cancer is dependent on the activities of E5, E6, and E7 proteins which are considered to be virally encoded oncoproteins (21, 32–35).

The cutaneous HPV5 and HPV8 E6 and E7 proteins also have oncogenic activities but they do not necessarily target the same tumor suppressor proteins as the high-risk mucosal HPV E6 and E7 oncoproteins (36–38). Specifically, the cutaneous HPV E6 oncoproteins do not directly target p53 but have been shown to interfere with the tumor suppressive NOTCH and TGF-β signaling pathways by interacting with the key transcriptional co-activators, MAML1 and SMAD2/SMAD3, respectively (39–42). By targeting these pathways, the cutaneous HPV E6 oncoproteins strongly inhibit keratinocyte differentiation (39–42). Cutaneous HPVs do not encode an E5 protein.

The murine papillomavirus MmuPV1 provides the unique opportunity to study papillomavirus-induced disease in a genetically tractable pre-clinical animal model (43–49). MmuPV1, like cutaneous HPVs, encodes for E6 and E7 proteins but does not encode for an E5 protein (50, 51). Previously, we have shown that MmuPV1 E7 targets similar cellular proteins as HPV E7, including the tumor suppressors pRB and PTPN14, and that these interactions contribute to viral pathogenesis (52–54). Interestingly, MmuPV1 E7 lacks a canonical LXCXE-based pRB binding site and interacts with a different region of pRB than high-risk HPV E7s, and, unlike the high-risk HPV E7s, MmuPV1 E7 does not promote E2F activity (52). MmuPV1 E6, like cutaneous HPV E6 proteins, inhibits the tumor suppressive NOTCH and TGF-β signaling pathways by interacting with MAML1 and SMAD2/SMAD3 (40). By targeting these pathways, MmuPV1 E6 inhibits keratinocyte differentiation (40). In addition, using MmuPV1 E6 mutant (R130A) that is defective for MAML1 binding, we found that the ability of MmuPV1 E6 to inhibit NOTCH signaling correlates with E6’s ability to contribute to viral pathogenesis (40). Additional work on MmuPV1 E6 has shown that E6 promotes basal cell identity and promotes proliferation post-confluency (55). This latter study also found that MmuPV1 E6’s interaction with LXXLL motif-containing proteins like MAML1 promotes these phenotypes through inhibition of differentiation (55). The work on MmuPV1 E6 has primarily focused on MmuPV1 E6’s ability to inhibit differentiation by targeting NOTCH signaling through interaction with MAML1. Collectively, these studies have provided compelling evidence that MmuPV1 E6 and E7 contribute to viral pathogenesis and revealed mechanistic insights into how they do so.

While cellular binding partners of MmuPV1 E6 and E7 have been identified and the roles of a subset of these interactions to viral pathogenesis have been delineated, it remains unclear specifically how the E6 and E7 oncoprotein alter the host cell to promote viral pathogenesis (40, 52, 53, 55). In this study, we specifically investigate how MmuPV1 E6 alters the host cell by individually expressing MmuPV1 E6 in mouse skin keratinocytes (MKs) *via* retroviral transduction, providing us the opportunity to potentially identify other activities of MmuPV1 E6 that contribute to viral pathogenesis beyond inhibition of keratinocyte differentiation. MmuPV1 E6 expressing MKs display a markedly increased proliferation rate and an ability to grow to higher density compared to the vector control MKs. We also found that MmuPV1 E6 expressing mouse keratinocytes outgrow the control vector transduced cells when cultured together and lose their sensitivity to growth-restrictive conditions. To determine potential mechanisms that drive the observed enhancement of proliferation, we subjected MmuPV1 E6 expressing MKs to quantitative proteomic and transcriptomic analyses to examine how MmuPV1 E6 may alter the host proteome and transcriptome, respectively. Both analyses identified E2F responsive genes being upregulated in MmuPV1 E6 expressing MKs, which we confirmed by qRT/PCR analyses. We also identified other cancer hallmarks not associated with the promotion of cell proliferation including evasion of growth suppressors, inhibition of immune response, resistance to cell death, and alterations in DNA damage response that are altered in MmuPV1 E6 expressing murine keratinocytes.

## RESULTS

### MmuPV1 E6 promotes proliferation of mouse keratinocytes *in vitro*

Primary mouse keratinocytes (MKs) were isolated from neonatal mice (prior to 4 days postpartum) as previously described (52, 53). Early passage MKs derived from multiple different mice were individually transduced with a pLXSN-based retroviral vector encoding MmuPV1 E6 or the parental pLXSN retrovirus not encoding E6. The expression of MmuPV1 E6 was confirmed by RT-PCR (Supplemental Figure 1). All subsequent experiments reported below were performed with MKs isolated from multiple different mice.

**Fig 1:**
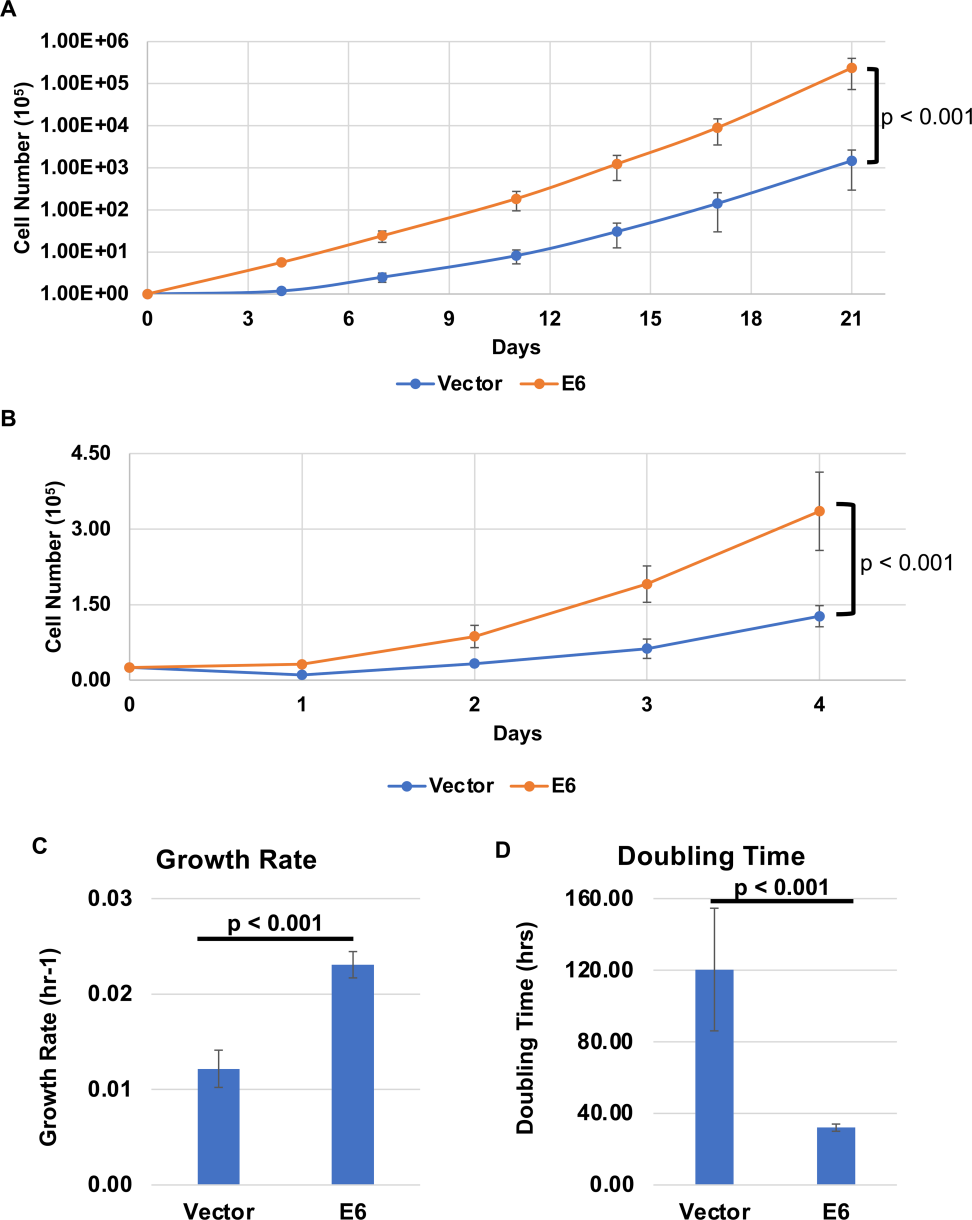
MmuPV1 E6 expressing mouse keratinocytes proliferate at a significantly faster rate. (**A**) MmuPV1 E6 expressing and vector control mouse keratinocytes were grown and continuously passaged over a 21-day period. Cells were counted and passaged every 3–4 days. Cell number was determined at each time point and plotted. Each condition was performed in biological triplicate and standard error is shown. A comparison of the Fits test was performed to determine statistical significance with the *P*-value shown. (**B**) MmuPV1 E6 and vector control cells were plated and grown over a 4-day period. Cells were counted daily and cell number determined over the 4-day period and plotted as shown. Each condition was performed in biological triplicate and standard error is shown. A comparison of the Fits test was performed to determine statistical significance with the *P*-value shown. The growth rate (**C**) and doubling time (**D**) of MmuPV1 E6 and vector control cells were calculated using the 21-day growth curve. At each time point (except Day 0), growth rate and doubling time were calculated leading to a total of 15 measurements which were used to calculate standard error and statistics. Wilcoxon rank-sum test was performed to determine statistical significance and the *P*-value is shown.

In deriving the MmuPV1 E6 transduced MKs, it was noted that they required more frequent passaging compared to the pLXSN vector-only (control) transduced MKs to maintain them as subconfluent cultures. This observation was reproducible in multiple experimental replicates across the MKs isolated from different mice. This led us to hypothesize that MmuPV1 E6 increases the rate of proliferation of MKs. To test this hypothesis, we plated 1 × 10^5^ cells per 6 cm dish and counted every 3–4 days for 21 days maintaining the cells under conditions of subconfluency. At each time point, MKs were trypsinized and counted using trypan blue staining, and 1 × 10^5^ cells per 6 cm dish were replated at each time point (days 0, 4, 7, 11, 14, 17, and 21) to maintain subconfluency. The number of MmuPV1 E6 expressing MKs accumulated at a significantly faster rate compared to the vector control cells (Fig. 1A). To determine whether this increase in cell number was due to an increase in the rate of cell proliferation versus a difference in plating efficiency, which could contribute to the differences observed above because the cells were passaged to maintain subconfluency, MmuPV1 E6 expressing MKs and vector control MKs were plated at 2.5 × 10^4^ cells per well in a six-well plate, and cell number was determined every day for 4 days. Cells were not counted beyond 4 days in these experiments because, by then, the E6 expressing MKs were reaching confluence. We observed that the MmuPV1 E6 expressing MKs proliferated at a significantly faster rate compared to the vector control cells during the short-term growth assay (Fig. 1B). The growth rate and doubling time of both the MmuPV1 E6 expressing and control vector MKs were calculated. The MmuPV1 E6 expressing MKs had a significantly higher growth rate and faster doubling time compared to the vector control cells (Fig. 1C and D). Collectively, these results show that MmuPV1 E6 expression increases the growth rate of mouse keratinocytes *in vitro*.

### MmuPV1 E6 also enhances the plating efficiency of mouse keratinocytes

**Fig 2:**
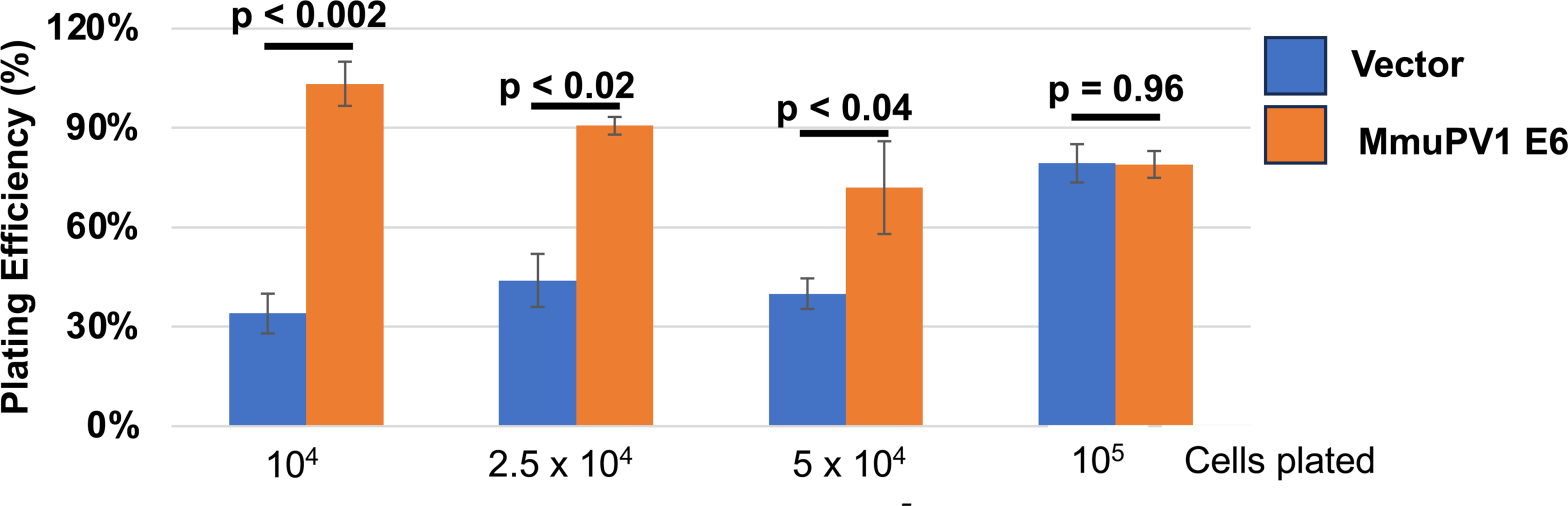
MmuPV1 E6 expressing MKs have increased plating efficiency at lower cell doses. MmuPV1 E6 expressing or vector control cells were plated at various densities (1 × 10^4^/0.01 cells/cm^2^, 2.5 × 10^4^/0.03 cells/cm^2^, 5 × 10^4^/0.05 cells/cm^2^, and 1 × 10^5^, 0.1 cells/cm^2^). Cells were counted ~18 hours later using trypan blue. Cell number was determined, and plating efficiency was calculated. The experiment was done in biological triplicate and a standard error was calculated. A *t*-test was performed for statistical significance and the *P*-value is shown.

An increase in cell numbers is dependent on a number of factors in *in vitro* cell culture including how efficiently the cells attach to plates after passaging. We found that 24 hours post-plating, there were a higher number of MmuPV1 E6 expressing MKs attached to the dish as compared to control vector transduced MKs (Fig. 1B). To more carefully assess the difference in plating efficiency, MmuPV1 E6 expressing and vector control MKs were plated at various cell densities on six-well plates because plating efficiency decreases as cells are diluted (56). MKs were trypsinized and counted at ~16 hours post-plating. This time point was chosen to allow us to determine cell number prior to significant levels of cell division based upon the doubling times determined in Fig. 1D. At lower cell densities, the MmuPV1 E6 expressing MKs adhered to the plate more efficiently than vector control transduced MKs; however, at high cell densities, this difference was lost (Fig. 2). Thus, in addition to increased cell division, MmuPV1 E6 expressing MKs can also show increased plating efficiency.

### MmuPV1 E6-expressing MKs outgrow vector control cells when passaged together

**Fig 3:**
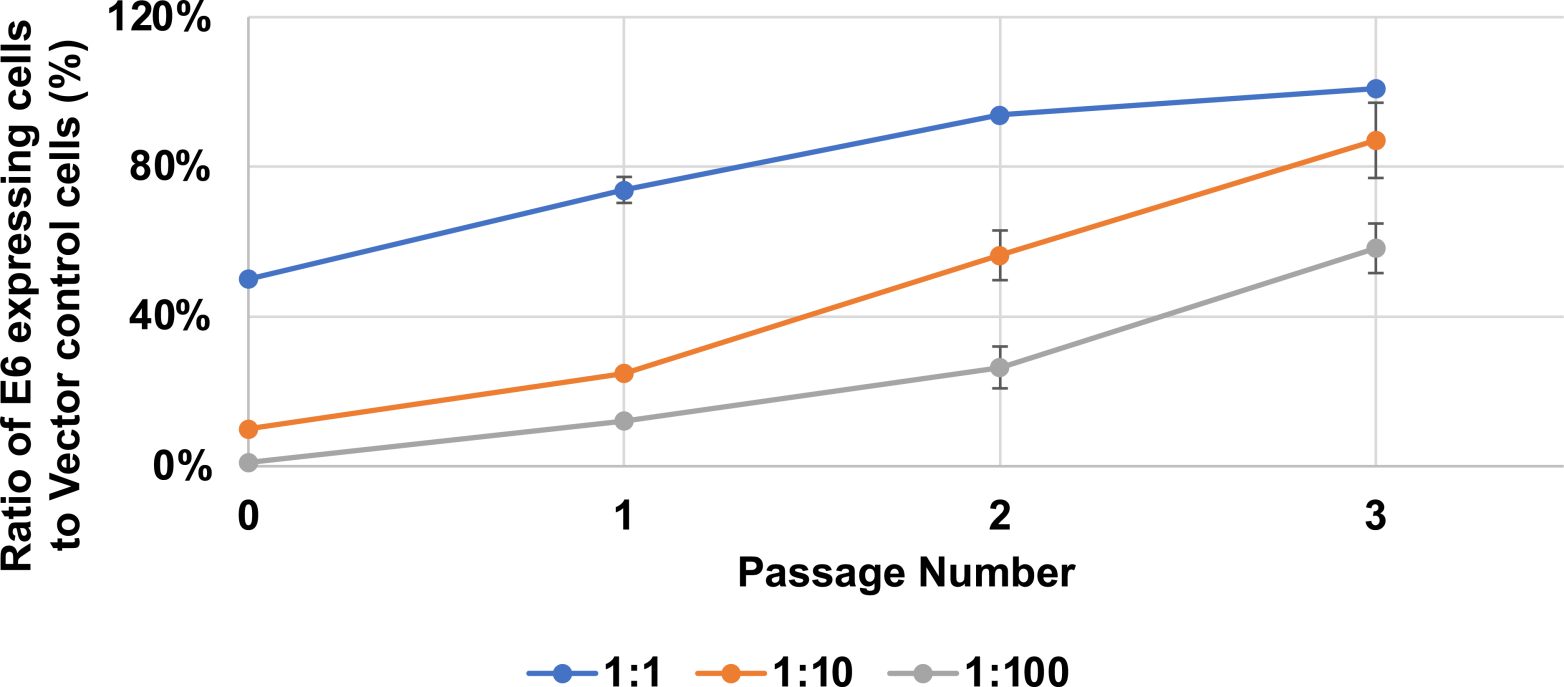
MmuPV1 E6 expressing MKs outgrow vector control cells in monolayer cell culture. MmuPV1 E6 and GFP co-expressing, and vector control cells were plated at various ratios including 1:1, 1:10, and 1:100 (E6:Vector), and allowed to grow for a period of three passages. Flow cytometry analysis was performed at each passage and percent MmuPV1 E6 expressing cells were determined and plotted as shown. The experiment was performed in biological triplicate and a standard error is shown.

Previous studies of high-risk HPV E6 expressing keratinocytes have shown that they outcompete their E6-negative counterparts (57). To determine whether the MmuPV1 E6 expressing MKs can outgrow the vector control MKs when co-cultured over multiple passages, we derived MmuPV1 E6 expressing MKs that express GFP by transducing them with a GFP expressing lentiviral vector under a different selection marker. Vector control cells were established by co-transducing retroviral and lentiviral control vectors. Cells were established and maintained under co-selection to ensure the expression of GFP. Following the establishment of cell strains, MmuPV1 E6-GFP expressing MKs were co-cultured with control vector transduced MKs at various ratios including 1:1, 1:10, and 1:100 (E6:vector) as previously described (58). A total of 1 × 10^5^ cells were plated per 6 cm dish. We monitored changes in the proportion of GFP+ cells compared to vector control cells over a period of three passages. The cells were maintained at subconfluency throughout. At each passage, cells were trypsinized, counted, and replated at 1 × 10^5^ cells per dish, and the remaining cells were subjected to flow cytometry analysis to determine the percentage of GFP+ cells. Prior to flow analysis, cells were stained with DAPI (4′,6-diamidino-2-phenylindole) to distinguish live from dead cells. Data were analyzed using the FlowJo software. The gating strategy and an example of a dot plot with GFP+ cells are shown in Supplemental Fig 2. The proportion of GFP+, E6 expressing MKs increased at each serial passage, with the 1:1 ratio reaching a nearly pure MmuPV1 E6 expressing population by the endpoint (Fig. 3). Thus, MmuPV1 E6 expressing MKs outgrow the vector control cells when co-cultured over multiple passages.

### MmuPV1 E6 expressing MKs can proliferate under growth-restrictive conditions

**Fig 4:**
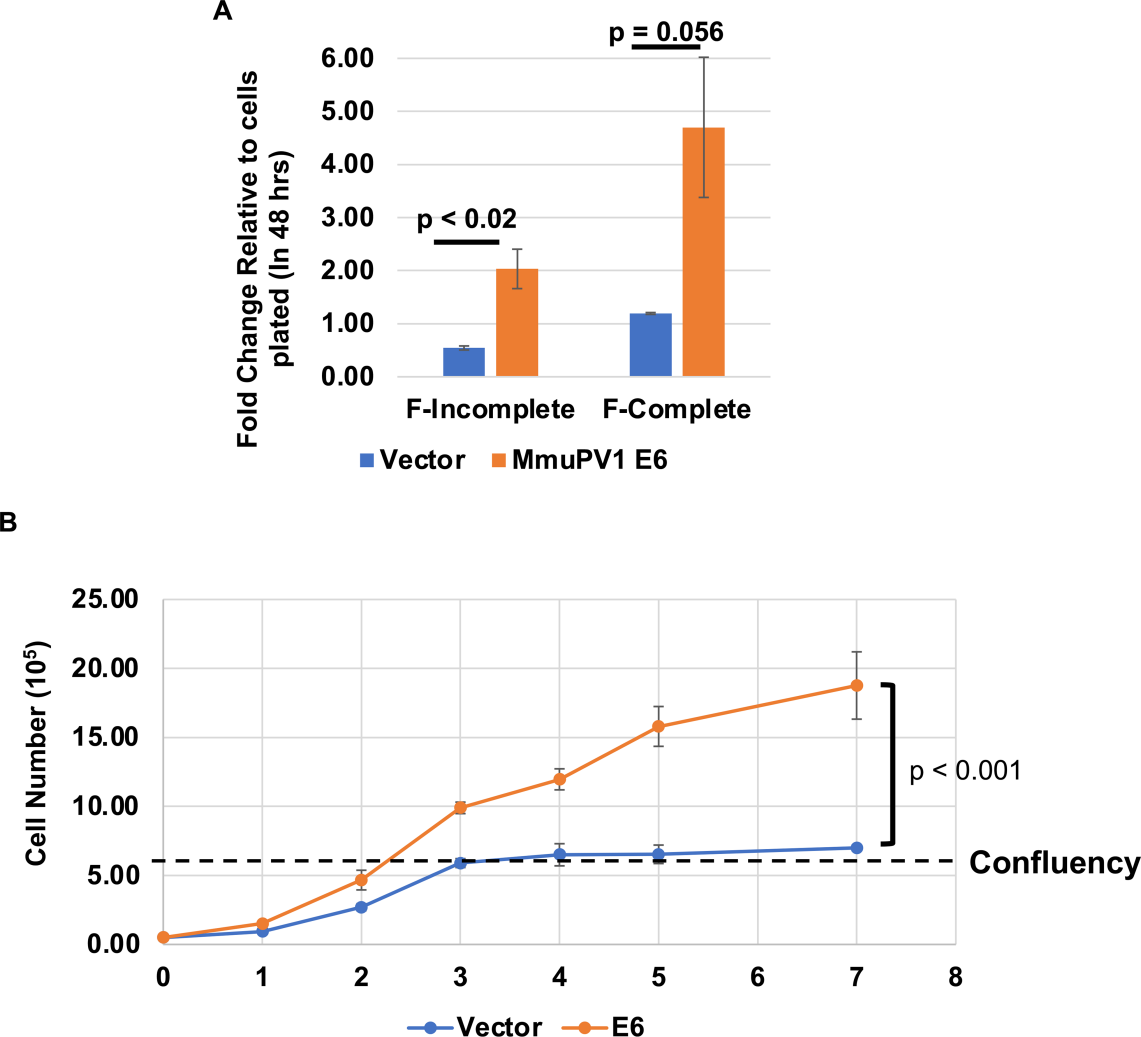
MmuPV1 E6 expressing mouse keratinocytes proliferate in growth-restrictive environments. (**A**) MmuPV1 E6 expressing and vector control mouse keratinocytes were plated and counted at 1, 2, 3, 4, 5, and 7 days post-plating. Cell number was calculated and plotted as shown. The red dashed line indicates when vector control cells became confluent. The experiment was performed in biological triplicate and a standard error is shown. (**B**) MmuPV1 E6 expressing and vector control cells were plated into F-complete or F-incomplete (F-media without epidermal growth factor, FBA, and insulin) and allowed to culture for 48 hours. At 48 hours, cells were counted using trypan blue stain, and cell number was calculated and plotted. The experiment was performed in biological triplicate, and a standard error is shown. A comparison of the Fits test was performed to determine statistical significance with the *P*-value as shown.

We have shown that MmuPV1 E6 expressing MKs grow at a faster rate and adhere to tissue culture plates more efficiently. We next asked whether they differ in their dependency on exogenous growth factors. The F-media used to culture the MKs in this study contains fetal bovine serum (FBS) as well as specific growth factors including epidermal growth factor (EGF) and insulin. To determine whether the MmuPV1 E6 expressing MKs require these growth factors to proliferate, we cultured MmuPV1 E6 expressing and control vector transduced MKs in F-media that lacked FBS, EGF, and insulin (referred to as F-incomplete). 1 × 10^5^ cells were plated in a 6 cm dish containing either F-incomplete or F-complete media and counted 48 hours post-plating. As expected, the control vector transduced MKs proliferated in F-complete media (Fig. 4A); however, in the F-incomplete media, the number of vector control cells was reduced compared to the initial number plated indicating that proliferation and survival of these cells was dependent on extrinsic growth factors (Fig. 4A). By contrast, the MmuPV1 E6 expressing MKs proliferated in both the F-complete and F-incomplete media, albeit to a lesser degree in the F-incomplete condition (Fig. 4A). The results from this experiment suggest that proliferation of MmuPV1 E6 expressing MKs is less dependent on extrinsic growth factors than control MKs.

To investigate whether MmuPV1 E6 expressing MKs can grow in another growth-restrictive environment, we asked whether the MmuPV1 E6 expressing MKs are resistant to contact inhibition. MmuPV1 E6 expressing MKs and control vector transduced MKs were plated and counted at six different time points post-plating (days 1–5 and day 7) without passaging them. We found that the vector control MKs reached confluency at 3 days post-plating and that the cell number did not increase after reaching confluency (Fig. 4B). By contrast, the MmuPV1 E6 expressing MKs reached confluency at 2 days post-plating and the cell number continued to increase past this point (Fig. 4B). These results indicate that the MmuPV1 E6 expressing MKs are resistant to contact inhibition and can grow to a higher density.

### MmuPV1 E6 significantly alters cellular homeostasis

**Fig 5:**
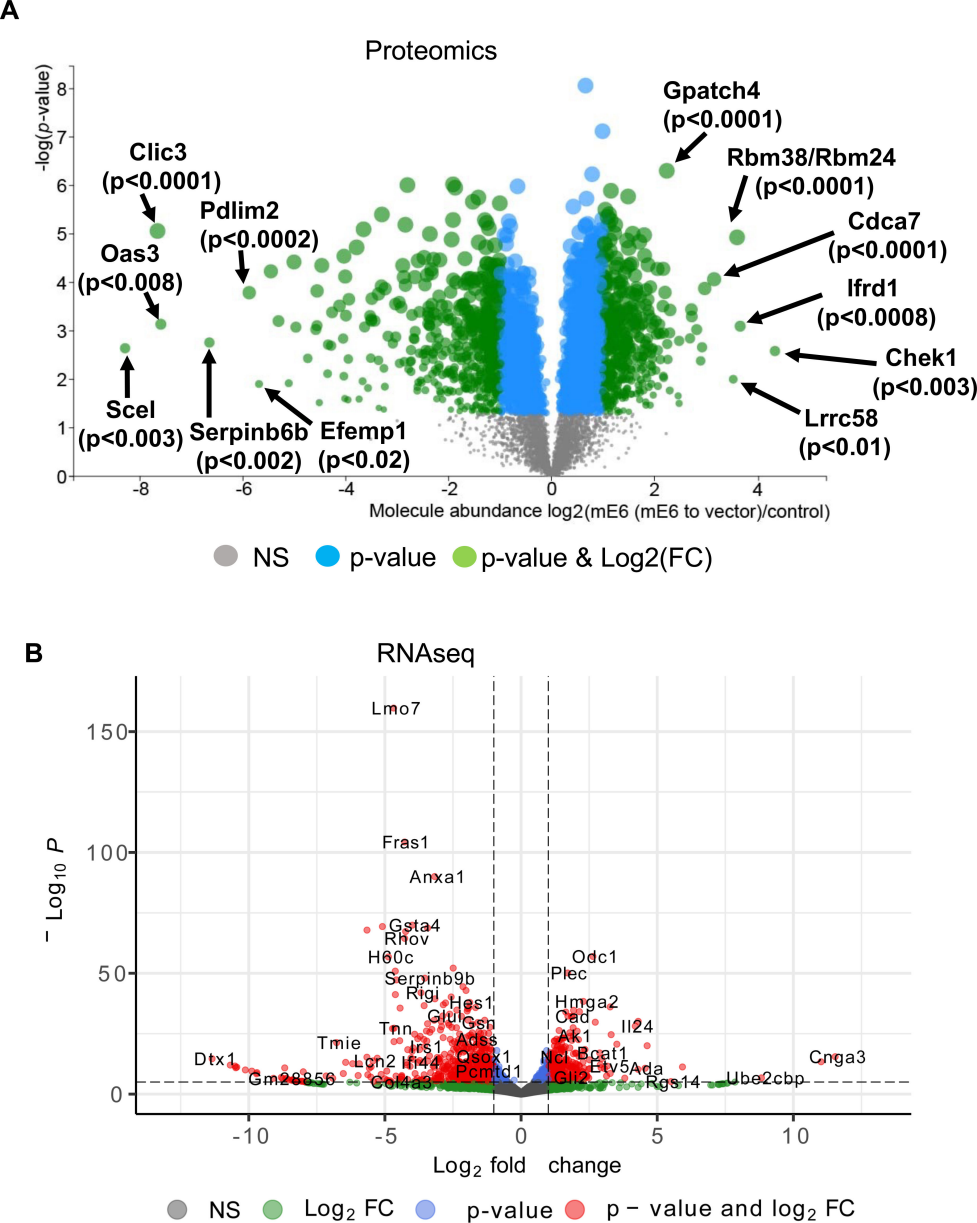
MmuPV1 E6 alters host cell proteome and transcriptome. Volcano plots for proteomic (**A**) and transcriptomic (**B**) analysis are shown. For proteomic data volcano plot (**A**), gray dots indicate no significance (NS), blue dots indicate *P*-value < 0.05, and green dots represent *P*-value < 0.05 and |Log2(FC)| >1. For transcriptomic data volcano plot, gray dot indicates not significant (NS), green indicate |Log2(FC)| >1, blue dots indicate *P*-value < 0.05, and red dots indicate *P*-value < 0.05 and |Log2(FC)| >1. A subset of genes that have a *P*-value < 0.05 and |Log2(FC)| >1 are indicated in each plot.

Our results show that MmuPV1 E6 promotes proliferation of keratinocytes but the potential mechanism by which MmuPV1 E6 promotes proliferation remains unclear. To start addressing this question, we performed proteomic and transcriptomic analysis of early passage MmuPV1 E6 expressing and vector control MKs through quantitative mass spectrometry using nanoflow liquid chromatography coupled with a hybrid quadrupole-Orbitrap-linear ion trap mass spectrometer (MS) and RNAseq analysis performed on ribodepleted total RNA on a NovaSeq, respectively (59). Following the selection of MmuPV1 E6 expressing and vector control cells, cell pellets were saved or lysed in trizol (to preserve RNA) at early passage (passage <5). The proteomic analysis was performed in a biological quadruplicate, that is, with 4-cell populations, each derived from an individual mouse. The RNAseq analysis was performed in biological triplicate, that is, with 3-cell populations, each derived from an individual animal. Our analysis of the MS data revealed that MmuPV1 E6 significantly altered the steady-state levels of ~900 cellular proteins (Log2(FC) ≥1 or Log2(FC) ≤ −1 and *P*-value ≤ 0.05). Proteins that showed the most significant upregulated or downregulated in our MS data spanned several different cellular processes including innate immune response (downregulated), keratinocyte differentiation (downregulated), DNA damage (upregulated), cellular proliferation (upregulated), and RNA biogenesis (upregulated) (Fig. 5). Similarly, our analysis of the RNAseq data revealed altered steady-state levels of transcripts associated with several different cellular processes including innate immunity (downregulated), NOTCH signaling (downregulated), antigen presentation (downregulated), polyamine syntheses (upregulated), and Hedgehog signaling (upregulated) (Fig. 5). Collectively, these results show that MmuPV1 E6 significantly alters the cellular proteome and transcriptome.

### MmuPV1 E6 increases the expression and abundance of genes associated with DNA replication and cellular proliferation

**Fig 6:**
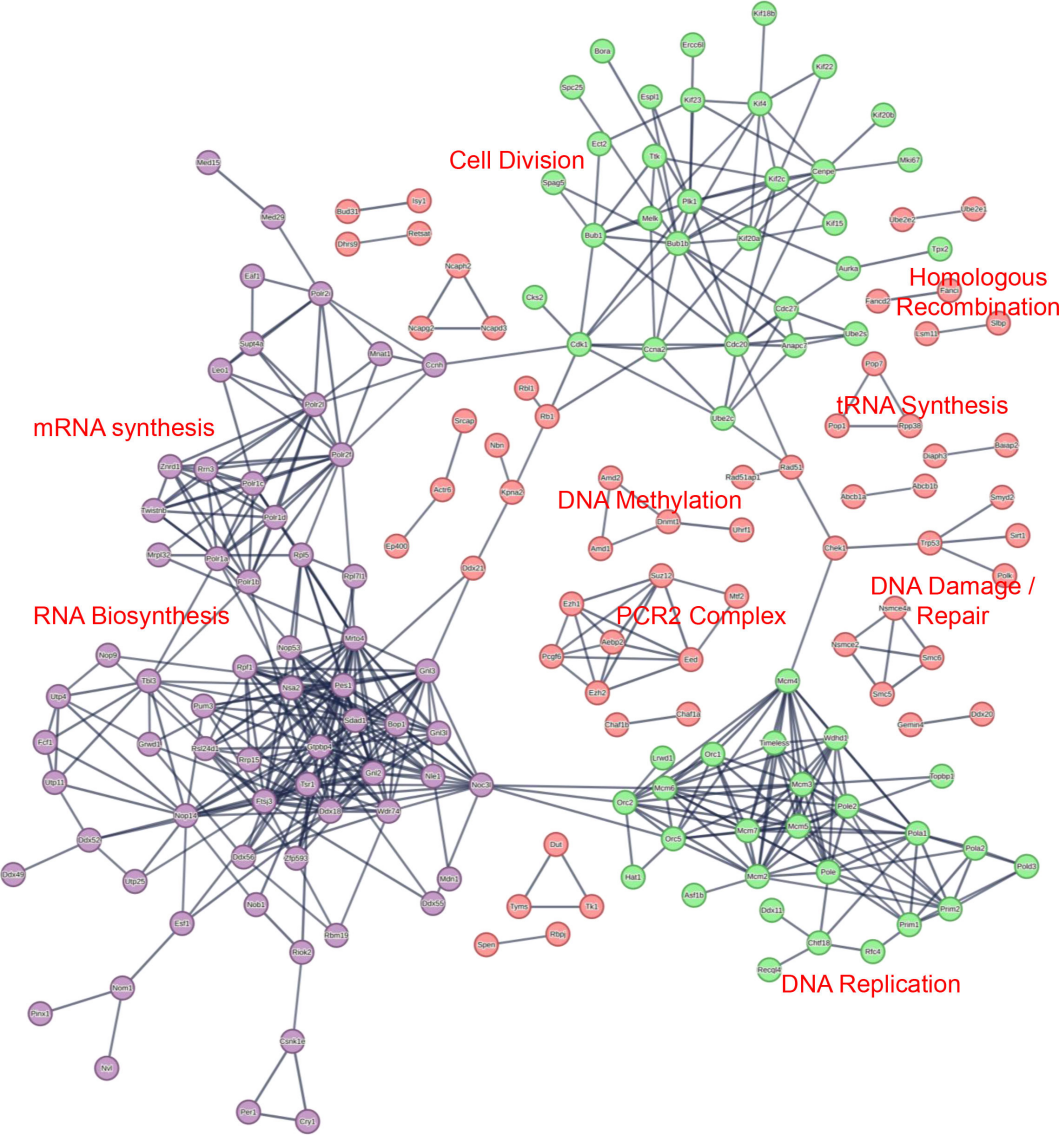
STRING network of significantly upregulated proteins in proteomics data. STRING network analysis of proteins with Log2(FC) >1 and *P*-value < 0.05 is shown. Specific clusters of proteins of interest are labeled within the image. Shared clusters between proteomic and RNAseq analysis are labeled in different colors including cellular proliferation in green and RNA synthesis in purple.

**Fig 7:**
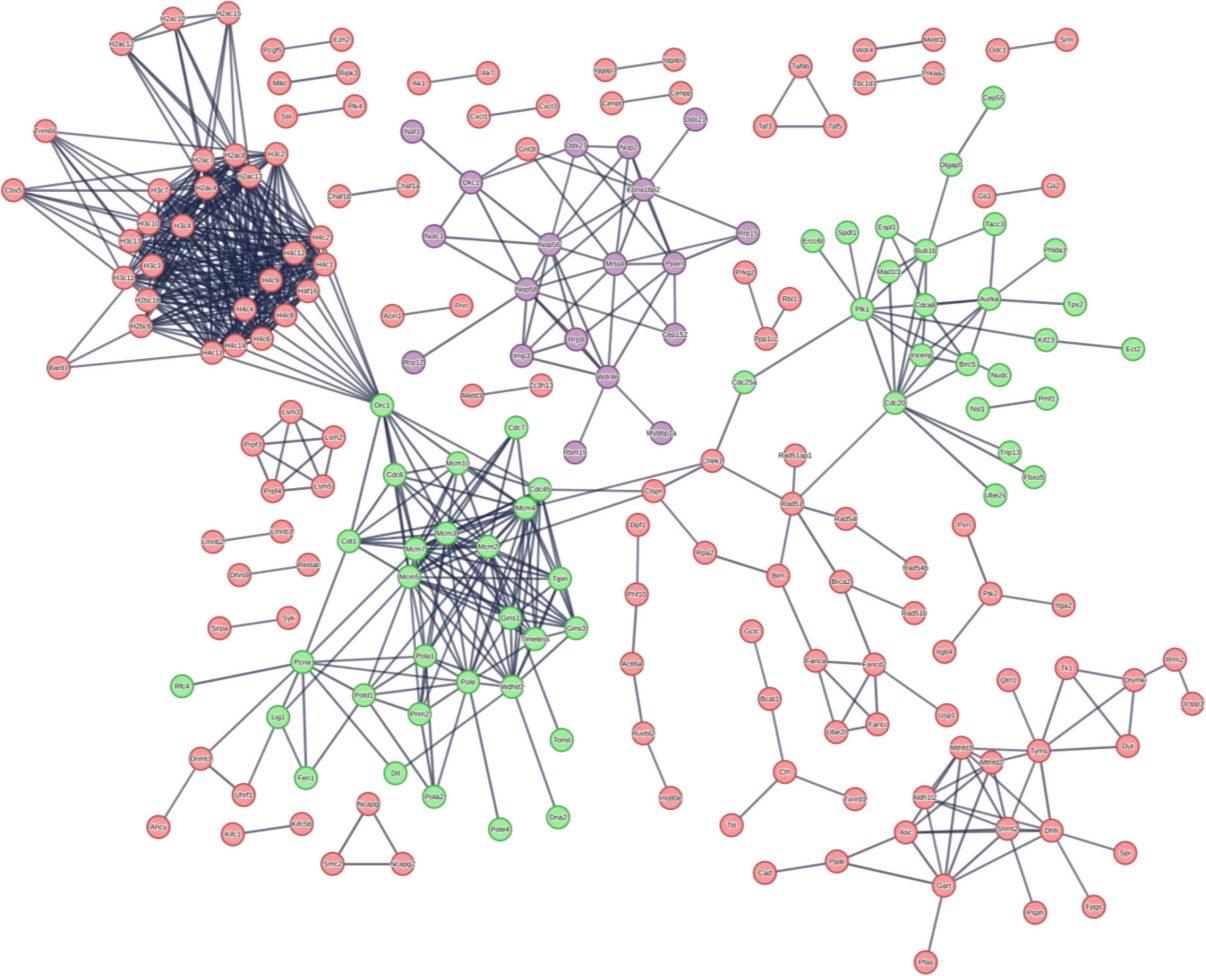
STRING network of significantly upregulated genes in transcriptomic data. STRING network analysis of genes with Log2(FC) >1 and *P*-value < 0.05 is shown. Specific clusters of proteins of interest are labeled within the image. Shared clusters between proteomic and RNAseq analysis are labeled in different colors including cellular proliferation in green and RNA synthesis in purple.

To understand how the various changes observed in Fig. 5 may alter specific cellular processes, the MS and RNAseq data were analyzed with various bioinformatics tools including STRING analysis, Gene Ontology (GO) analysis, and Gene Set Enrichment Analysis (GSEA). For the MS data, the proteins that had a |Log2(FC)| >1 or *P*-value < 0.05 were subjected to STRING and GO analysis. Separate STRING analyses were performed for upregulated and downregulated proteins to generate protein interaction networks. STRING analysis of the upregulated proteins in our MS data revealed a number of clusters associated with cell division/proliferation, DNA replication, mRNA synthesis, and RNA biosynthesis which may be associated with the increase in cellular proliferation we observed in our MmuPV1 E6 expressing MKs *in vitro* (Fig. 6). Interestingly, STRING analysis of significantly upregulated genes in our RNAseq data revealed similar results with clusters associated with DNA replication, cell division, histones, nucleotide synthesis, and tubulin, which also could be tied to the increase in cellular proliferation (Fig. 7). We also observed that E6 caused an increase in protein and mRNA levels of genes known to play a role in DNA damage response and replication stress (Fig. 6 and 7).

**Fig 8:**
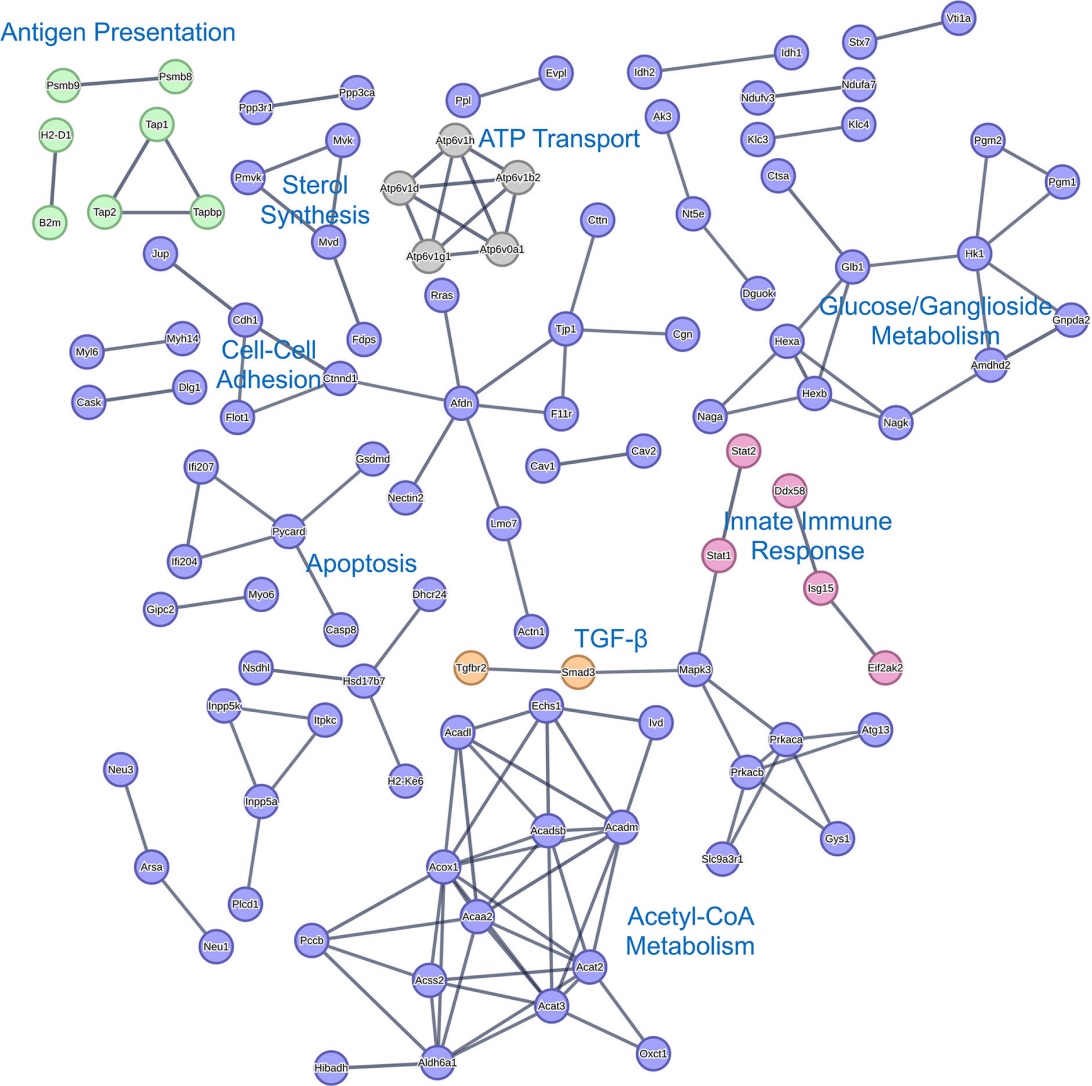
STRING network of significantly downregulated proteins in proteomics data. STRING network analysis of proteins with Log2(FC) < −1 and *P*-value < 0.05 is shown. Specific clusters of proteins of interest are labeled within the image. Shared clusters between proteomic and RNAseq analysis are labeled in different colors including antigen presentation in green, TGF-β in orange, innate immune response in pink, and ATP transport in gray.

**Fig 9:**
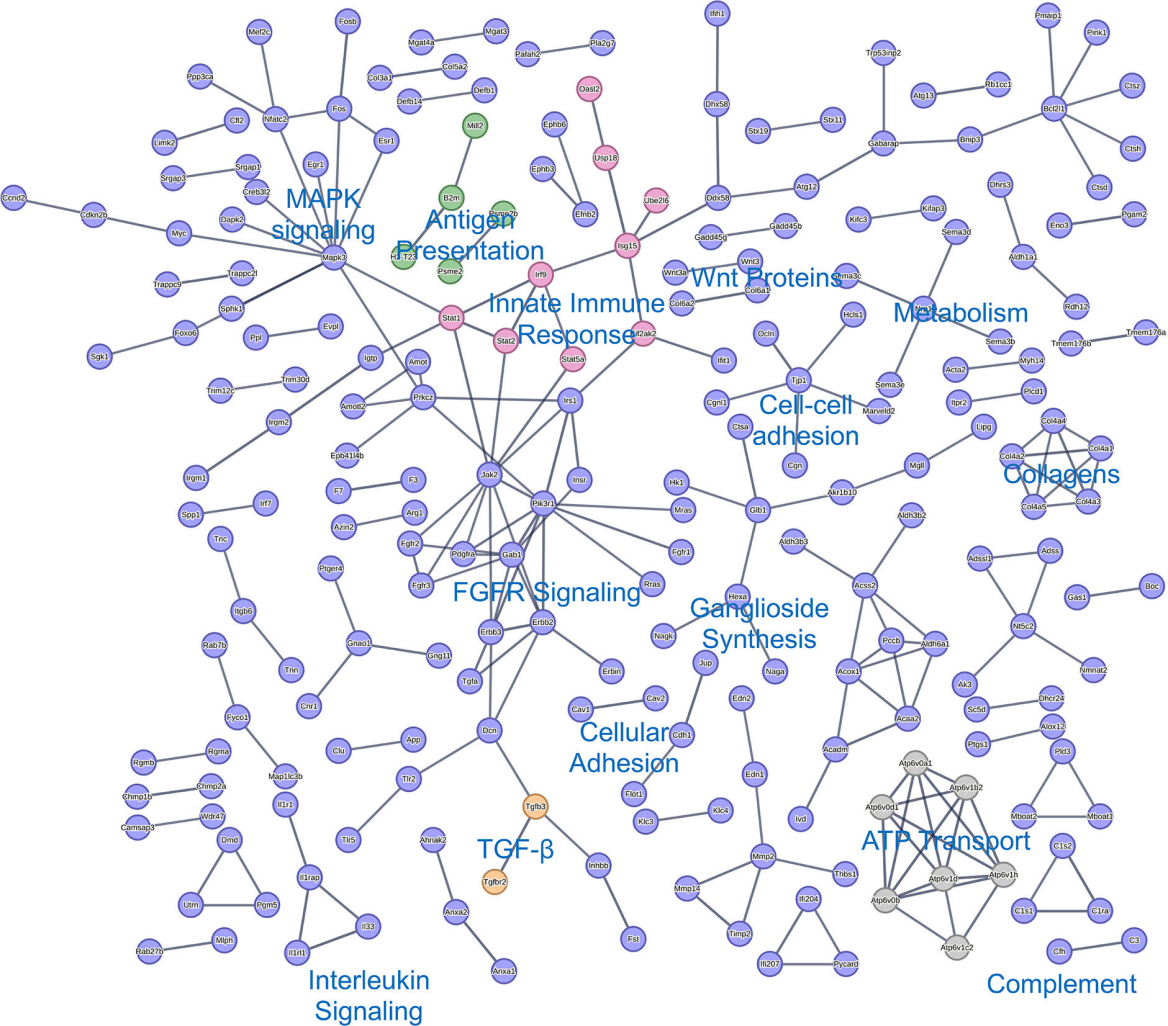
STRING network of significantly downregulated genes in transcriptomic data. STRING network analysis of genes with Log2(FC) < −1 and *P*-value < 0.05 is shown. Specific clusters of proteins of interest are labeled within the image. Shared clusters between proteomic and RNAseq analysis are labeled in different colors including antigen presentation in green, TGF-β in orange, innate immune response in pink, and ATP transport in gray.

STRING analysis of the significantly decreased proteins and mRNAs indicates that MmuPV1 E6 inhibits a variety of cellular processes. STRING analysis of the qMS data showed a negative enrichment of proteins associated with TGF-β signaling, the innate immune response, antigen presentation, several metabolic processes, cell-cell interactions, and ATP transport (Fig. 8). STRING analysis of the RNAseq data revealed overlap in the cellular processes perturbed by MmuPV1 E6 including the innate immune response, antigen presentation, metabolic processes, and ATP transport (Fig. 9). In addition, the expression of WNT genes, interleukin signaling, MAPK, and FGFR signaling was inhibited at the RNA level but was not observed in the MS data analyses (Fig. 9). The inhibition of these various pathways provides insight into potential mechanisms by which MmuPV1 E6 promotes the *in vitro* phenotypes we have observed including less dependence on growth factors (reduced MAPK signaling).

**Fig 10:**
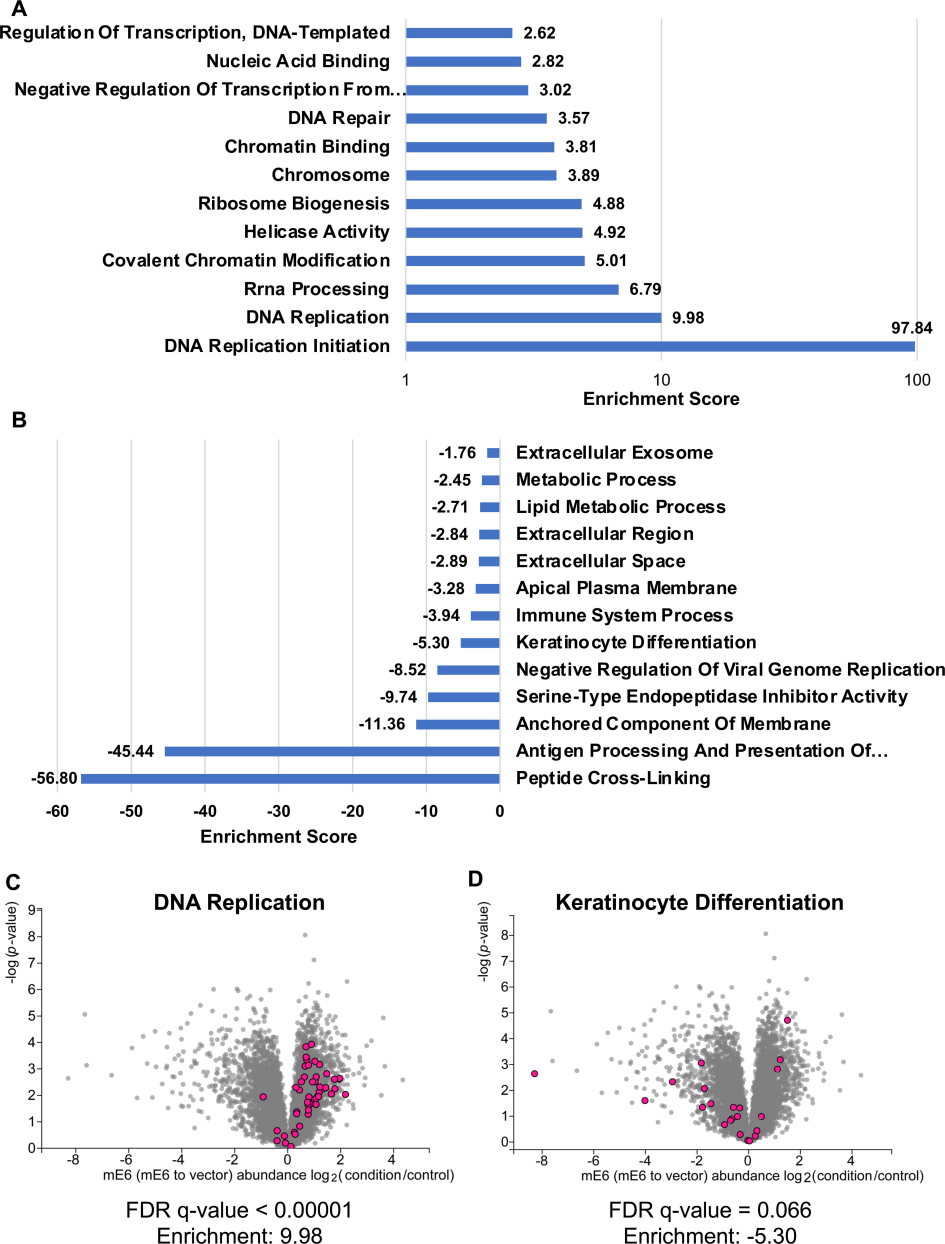
Gene ontology analysis suggests that MmuPV1 E6 promotes cellular proliferation. (**A**) The top 10 gene sets that are positively enriched in MmuPV1 E6 expressing MKs from gene ontology analysis of the proteomics data are shown. (**B**) The top 10 gene sets that are negatively enriched in the MmuPV1 E6 expressing MKs from gene ontology analysis of the proteomics data are shown. The enrichment score for each gene set is shown in both (**A**) and (**B**). (**C**) A volcano plot of the proteomics data is shown with proteins associated with the DNA replication gene set from the gene ontology analysis. The pink dots indicate proteins present within the gene set. (**D**) A volcano plot of the proteomics data is shown with proteins associated with the keratinocyte differentiation gene set from the gene ontology analysis. The pink dots indicate proteins present within the gene set.

In addition to STRING analysis, we subjected our MS data to GO analysis to determine whether there were specific signaling pathways that could be correlated with the increase in cellular proliferation. GO analysis was performed separately on the significantly increased (Log2(FC) >1 and *P*-value < 0.05) and decreased (Log2(FC) < −1 and *P*-value < 0.05) proteins. Among the significantly increased GO terms were ones associated with DNA replication, RNA synthesis, and cell division, which is consistent with our STRING analysis of the significantly upregulated proteins (Fig. 10A). The significantly decreased GO terms were primarily associated with the innate immune response, metabolic processes, keratinocyte differentiation, and cellular anchorage (Fig. 10B). We generated volcano plots for the keratinocyte differentiation (decreased) and DNA replication gene sets (increased) (Fig. 10C and D). In the DNA replication volcano plot, we see that the majority of the proteins within this GO term are upregulated in the MmuPV1 E6 expressing MKs (Fig. 10C). By contrast, the proteins within the keratinocyte differentiation GO term are predominantly downregulated in the MmuPV1 E6 expressing MKs, consistent with previous studies (Fig. 10D) (40).

**Fig 11:**
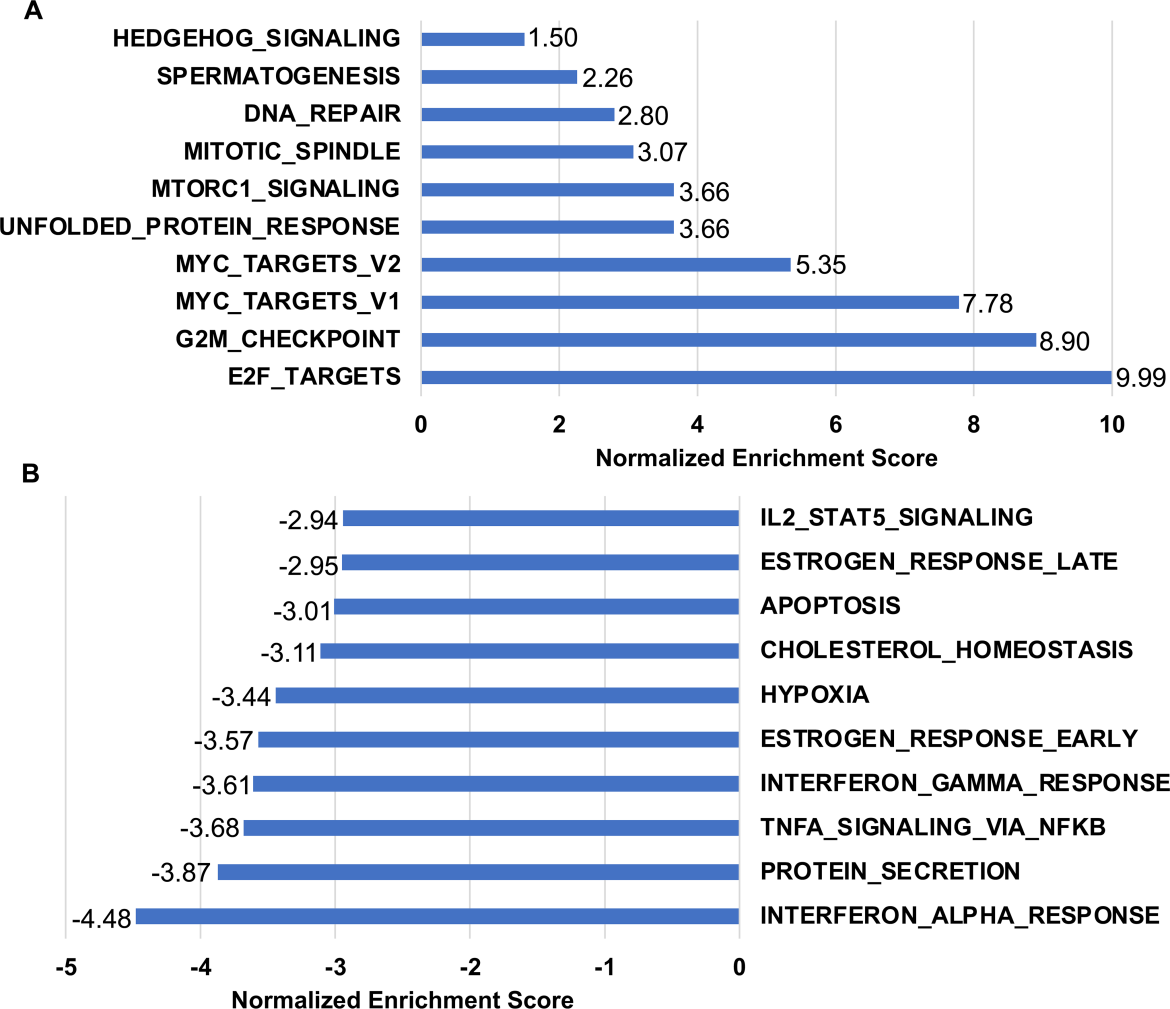
GGSEA suggests that MmuPV1 E6 perturbs a number of cellular pathways. The top 10 most significantly positively (**A**) and negatively (**B**) enriched gene sets from GSEA are shown. All gene sets had an FDR q-value < 0.05 and a normalized enrichment score is shown for each gene set.

**Fig 12:**
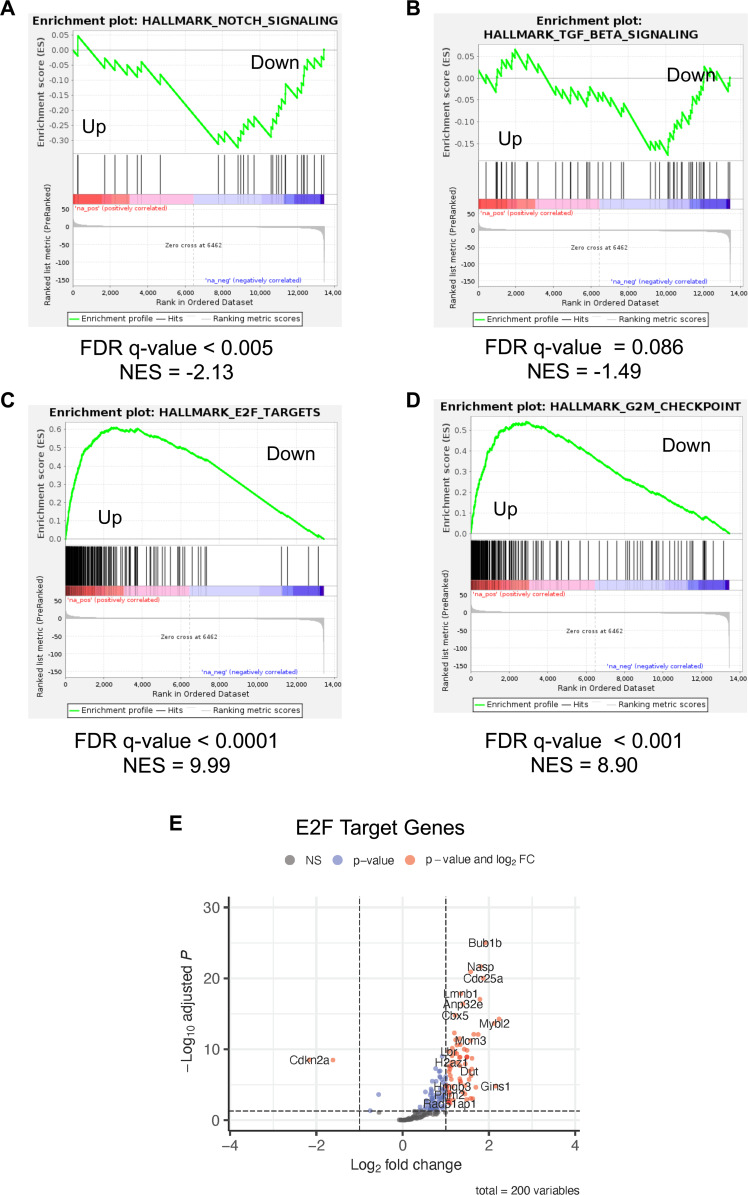
GSEA analysis reveals that MmuPV1 E6 expression increases E2F gene transcripts. GSEA plots for various signaling pathways are shown. HALLMARK_NOTCH_SIGNALING (**A**) and HALLMARK_TGF_BETA_SIGNALING (**B**) are shown which are known signaling pathways that are inhibited by MmuPV1 E6 and the majority of genes are negatively enriched (Down) in MmuPV1 E6 expressing mouse keratinocytes. GSEA plots for HALLMARK_E2F_TARGETS (**C**) and HALLMARK_G2M_CHECKPOINT (**D**) gene sets are shown. Most of the genes are positively enriched (Up) in MMuPV1 E6 expressing mouse keratinocytes. (**E**) A volcano plot of E2F target genes is shown where the majority of genes are upregulated with a subset of the genes indicated on the plot.

**Fig 13:**
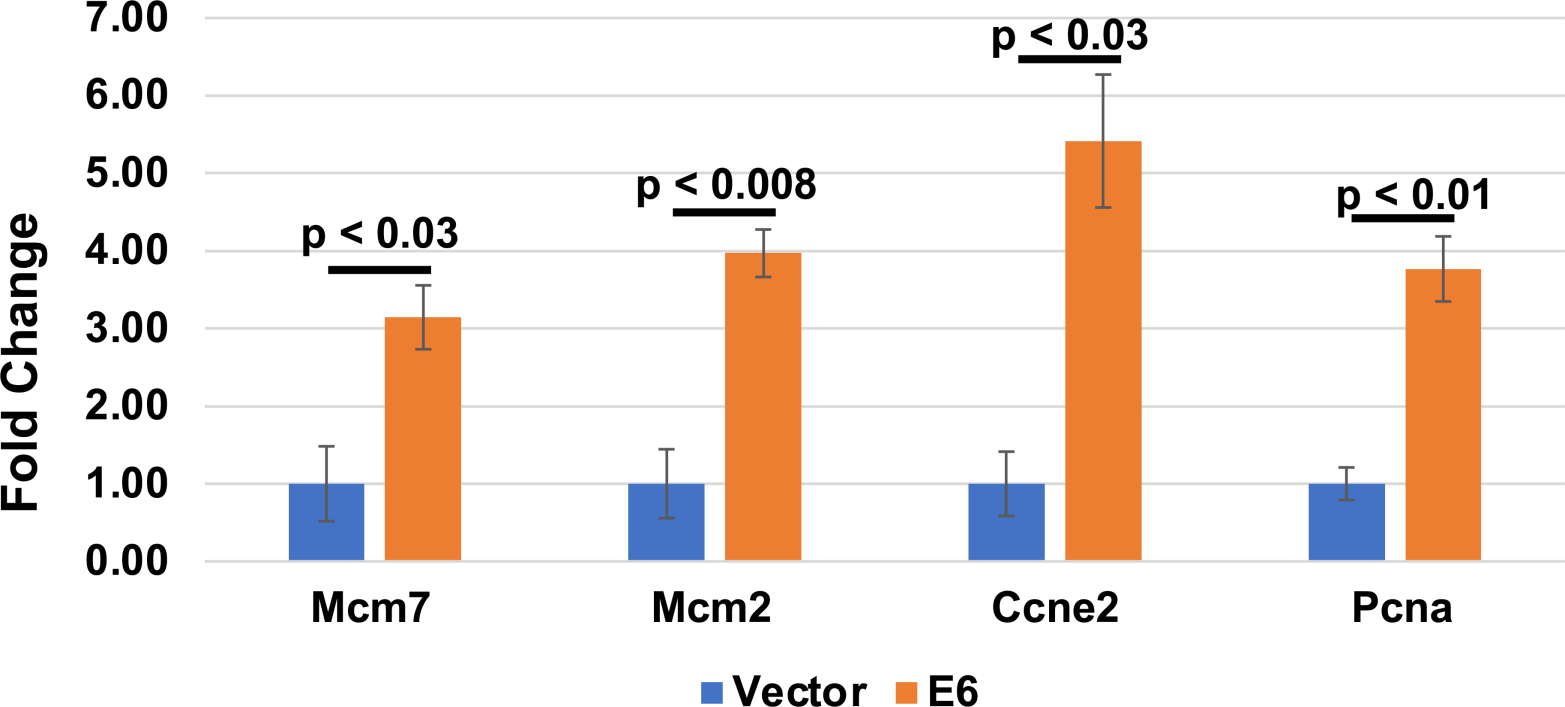
E2F target gene transcripts are elevated in MmuPV1 E6 expressing mouse keratinocytes. RNA was isolated from vector and MmuPV1 E6 expressing mouse keratinocytes and cDNA generated from the RNA. qRT-PCR was performed for the E2F target genes Mcm7, Mcm2, Ccne2, and Pcna. Fold change in transcript levels is shown. The experiment was performed in biological triplicate and a standard error is shown. A,t-test was used for statistical analysis and the *P*-value is shown.

We also subjected our RNAseq data to GSEA to determine which cellular pathways are altered in MKs expressing MmuPV1 E6. When we focus on the 10 most significantly enriched hallmark gene sets in our GSEA of the RNAseq, we observed that a number of gene sets associated with DNA replication and cellular proliferation are positively enriched in the RNAseq data including E2F_TARGETS, G2M_CHECKPOINT, MYC_TARGETS_V1, MYC_TARGETS_V2, MITOTIC_SPINDLE, and SPERMATOGENESIS (Fig. 11A, 12C and D). We also observed increases in the abundance of proteins transcriptionally regulated by E2F (Table 1). Also consistent with our proteomic analyses, we observed a significant positive enrichment of genes associated with DNA_REPAIR pathway (Fig. 11A). There was also upregulation cellular signaling pathways in the top 10 most positively enriched gene sets including MTORC1_SIGNALING and HEDGEHOG_SIGNALING (Fig. 11A). Consistent with our observations in the STRING and proteomic analysis, we observed a significant negative enrichment of genes associated with the innate immune response pathways including the gene sets for INTERFERON_ALPHA_RESPONSE, TNFA_SIGNALING_VIA_NFKB, INTERFERON_GAMMA_RESPONSE, and ILS_STAT5_SIGNALING (Fig. 11B) in the top 10 most downregulated gene sets. There were a number of other cellular pathways that were negatively enriched in our GSEA analysis of the RNAseq data including gene sets for APOPTOSIS, HYPOXIA, P53_PATHWAYS, EPITHELIAL_MESECHYMAL_TRANSITION, and APICAL_JUNCTION (Fig. 11B**,** 12A and B**;** Fig S3). Consistent with our previous work, the GSEA analyses show that there is a negative enrichment in both the NOTCH (NES = −2.13) and TGF-β (NES = −1.49) signaling pathways (Fig. 12A and B) (40). The GSEA of the RNAseq data showed a significant positive enrichment of E2F target gene transcripts and G2M checkpoint genes (Fig. 12C and D). When we plotted the E2F target genes on a volcano plot, most of the genes within this gene set were upregulated in the MmuPV1 E6 expressing MKs with the majority being significantly upregulated including Bub1b, Mcm3, Csc25a, Nasp, Lmnb1, Mybl2, and Gins1 (Fig. 12E). There is a small subset of the genes in the HALLMARK_E2F_TARGET gene set that was downregulated including Cdkn2A, which can encode p16^Ink4a^ and p19^Arf^, being most significantly downregulated. We confirmed the increase in E2F-responsive genes by performing qRT-PCR on well-described E2F responsive Mcm7, Mcm2, Ccne2, and Pcna genes from RNA isolated from early passage MmuPV1 E6 expressing MKs compared to vector control MKs. Consistent with our RNAseq analysis, we found significant increases (threefold–fivefold) in the expression of these E2F responsive genes in the MmuPV1 expressing MKs (Fig. 13). Collectively, our data suggest that MmuPV1 E6 promotes cellular proliferation and DNA replication at least in part through increased expression of E2F-responsive genes.

**TABLE 1:** E2F responsive genes associated with DNA replication from proteomics data

Gene	Fold change	*P-*value
Mcm2	2.28	0.0075
Mcm3	2.10	0.0220
Mcm4	2.34	0.0111
Mcm5	2.10	0.0131
Mcm6	2.19	0.0111
Pola1	2.79	0.0015
Pola2	2.45	0.0034
Pold3	2.45	1.98E-04
Ccna2	2.83	0.0466

### MmuPV1 E6’s interaction with LXXLL motif-containing cellular proteins significantly contributes to the proliferation of phenotypes *in vitro*

**Fig 14:**
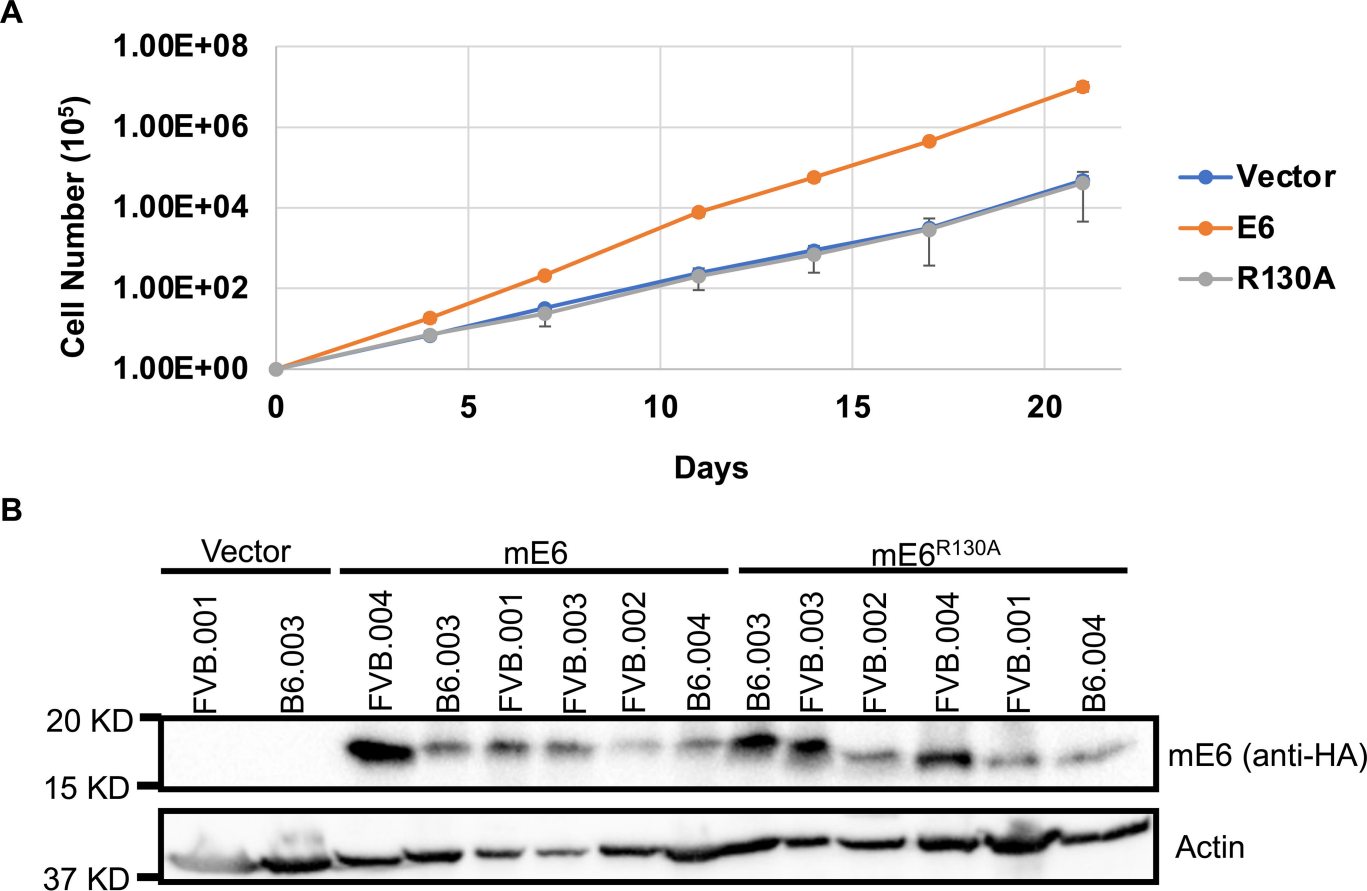
MmuPV1 E6’s interaction with LXXLL motif proteins significantly contributes to proliferation phenotype. (**A**) MmuPV1 E6- and MmuPV1 E6^R130A^ expressing cells and vector control cells were grown and continuously passaged over a 21-day period to maintain subconfluency. Keratinocytes were counted and passaged every 3 to 4 days. Fold change in cell number was determined, and a cumulative increase in cell number was calculated and plotted. The experiment was performed in biological duplicate and a standard error is shown. A comparison of the Fits test was performed to determine statistical significance; MmuPV1 E6 vs vector *P* < 0.0001, MmuPV1 E6^R130A^ vs vector *P* = 0.797, and MmuPV1 E6 vs MmuPV1 E6^R130A^ *P* < 0.0001. (**B**) MmuPV1 E6 is HA tagged and Western blot analysis was used to confirm expression of MmuPV1 E6 and MmuPV1 E6^R130A^. Western blot analysis is shown using antibodies against HA (MmuPV1 E6) and actin.

We have previously established that MmuPV1 E6 interacts with LXXLL motif-containing proteins including the transcriptional activator of NOTCH signaling, MAML1, and that these interactions play a critical role in viral pathogenesis (40). In addition, work from the Doorbar lab connected MmuPV1 E6’s ability to promote basal cell identity to the presence of an intact binding site for LXXLL proteins on MmuPV1 E6 (55). To determine whether MmuPV1 E6’s interaction with LXXLL motif-containing proteins including MAML1 contributes to E6’s proliferation phenotype *in vitro*, growth curve analysis was performed on mouse keratinocytes expressing HA-tagged MmuPV1 E6 or MmuPV1 E6^R130A^ transduced using lentiviral expression vectors. Cells were maintained subconfluent and were counted and passaged every 3 to 4 days. The MmuPV1 E6 expressing MKs proliferated at a significantly higher rate compared to the vector control cells, similar to what we observed in Fig. 1 (Fig. 14A). Interestingly, the MmuPV1 E6^R130A^ mutant expressing MKs proliferated at a rate similar to the vector control expressing MKs and significantly slower than the wild-type MmuPV1 E6 expressing mouse keratinocytes (Fig. 14A). We performed immunoblot analysis of E6 expression in vector control, MmuPV1 E6, and MmuPV1 E6^R130A^ expressing mouse keratinocytes to confirm the expression of the wild-type E6 and E6^R130A^ proteins. We found that the MKs expressed the MmuPV1 E6 and E6^R130A^ mutant at similar levels (Fig. 14B). These results suggest that MmuPV1 E6’s interaction with LXXLL motif-containing proteins such as MAML1 significantly contributes to the *in vitro* proliferation phenotype.

## DISCUSSION

In this study, we have observed that MmuPV1 E6 promotes cellular proliferation and increases the growth rate of MKs *in vitro* when maintained as subconfluent cultures (Fig. 1). This increase in proliferation correlated with an enhancement in plating efficiency of MKs when plated at low density (Fig. 2). In addition, MmuPV1 E6 expressing MKs outgrew vector control cells when co-cultured together over multiple passages (Fig. 3). MmuPV1 E6 also enhanced the ability of MKs to grow under conditions of contact inhibition and limiting growth factors (Fig. 4). Our observations are *in vitro* correlates to two of the major hallmarks of cancer, “sustaining proliferative signaling” and “evading growth suppressors” (60). Through unbiased quantitative mass spectrometry (MS) and RNAseq analyses, we found that MmuPV1 E6 significantly alters the homeostasis of hundreds of cellular genes and proteins (Fig. 5). To determine how these alterations contribute to an increase in proliferation, we performed STRING analysis, GO analysis, and GSEA to determine which cellular processes may contribute to the increase in proliferation. Collectively, these analyses indicate that MmuPV1 E6 promotes the increased accumulation and transcription of a number of cellular genes and their gene products that play roles in DNA replication and cellular proliferation (Fig. 6 to 12), consistent with our experimental data in cell culture (Fig. 1 to 5). Among these were multiple E2F-responsive genes, which we confirmed were upregulated by qRT-PCR (Fig. 12 and 13). Interestingly, we found that MmuPV1 E6’s interaction with LXXLL motif-containing proteins such as MAML1 significantly contributes to MmuPV1 E6’s ability to promote cell proliferation *in vitro* as the E6^R130A^ mutant defective for binding LXXLL motif-containing cellular proteins grew at a similar rate as vector control cells (Fig. 14). Further work is needed to determine whether an intact binding site for cellular LXXLL proteins is required for other *in vitro* phenotypes that we describe here. In addition, it is necessary to determine which E6-interacting cellular LXXLL motif-containing protein specifically contributes to the observed proliferation phenotype. How MmuPV1 E6 is increasing E2F activity also remains unclear and warrants further investigation.

The enhanced proliferation of MmuPV1 E6 allows the cells that express MmuPV1 E6 to outgrow their vector control counterparts when co-cultured and maintained in a subconfluent state (Fig. 3). The assays we performed are different from the competition assays performed with HPV16 E6, which examined colony outgrowth in confluent cell culture (57). We did consistently observe small GFP-positive islands of cells when we co-cultured MmuPV1 E6 expressing MKs and vector control MKs at the 1:100 ratio but did not follow these islands in a post-confluent state. Therefore, it remains to be determined whether MmuPV1 E6 expressing MKs will outcompete vector control cells as in the previously published competition assays for HPV16 E6.

For high-risk HPVs, induction of E2F-responsive genes is achieved by high-risk HPV E7’s ability to interact and promote degradation of the tumor suppressor pRB, which is mediated by high-risk HPV E7’s LXCXE motif that binds to the “pocket” domain of pRB (15, 34, 35, 61, 62). Our previous work found that MmuPV1 E7, which lacks an LXCXE motif, nevertheless can interact with pRB; however, MmuPV1 E7 does not promote degradation of pRB, nor does it lead to increased E2F activity (52). In our previous work, we found that MmuPV1 mutant virus that encodes E7 mutants that are defective for binding pRB or PTPN14 still show the induction of E2F responsive genes (52, 53). This leads us to hypothesize that MmuPV1 E7 may not increase cell proliferation. Certain HPVs encode E7 proteins that also lack an LXCXE motif, and bind pRb through their C terminus, like MmuPV1 E7, and engage a domain in Rb known to confer non-canonical pRB activities not associated with regulation of cell proliferation, all of which suggests that the binding to pRB by these E7 proteins may modulate activities of pRB that are not related to driving cell proliferation unless the E7 can promote degradation of pRB (63–71). This leads us to predict that the E6 proteins encoded by these HPVs, like MmuPV1 E6, drive cell proliferation and enhance expression of E2F-responsive genes, instead of their E7 proteins.

MmuPV1 E6 expression in MKs renders the cells resistant to growth-restrictive environments such as contact inhibition and reduced growth factors in the media. We observed that MmuPV1 E6 expressing MKs continue to increase in cell number post-confluency (Fig. 4B). Doorbar and colleagues have performed studies of MmuPV1 E6 protein in human keratinocytes, rather than mouse keratinocytes (55). They also observed that MmuPV1 could override cell contact inhibition in human keratinocytes and they found this property to correlate with MmuPV1 E6’s ability to bind LXXLL motif-containing proteins such as MAML1 (55). Their study also provided evidence that MmuPV1 E6 promotes basal cell identity (55). Our previous work on MmuPV1 E6 revealed that MmuPV1 E6’s ability to interact with LXXLL motif-containing proteins such as MAML1 is critical for its ability to inhibit differentiation *in vitro* and for MmuPV1 to cause pathogenesis *in vivo* (40). In addition, high-risk HPV 16 E6 also promotes post-confluent proliferation in human keratinocytes through inactivation of p53 and NOTCH signaling and potentially represents a shared activity of MmuPV1 E6 and high-risk HPV E6 (72). Based on the studies with human keratinocytes (40) alongside our observations in mouse keratinocytes, a plausible hypothesis arises: the ability of MmuPV1 E6 to inhibit differentiation and promote basal cell identity appears to be mediated, at least in part, by the presence of an intact bonding site for cellular LXXLL protein on MmuPV1 E6.

We found that MmuPV1 E6 expressing MKs are less dependent on exogenous growth factors to sustain cellular proliferation (Fig. 4A). Our results are consistent with previous observations with high-risk HPV 16 E6 expressing human keratinocytes and provide evidence for another shared activity between MmuPV1 E6 and high-risk HPV E6 proteins (72). Based on our proteomic and transcriptomic data, we posit that this may be driven by MmuPV1 E6’s induction of pro-survival signaling pathways such as MTOR signaling as evidenced by the GSEA analysis and its reduction of pro-apoptotic and growth-restrictive pathways, including the TGF-β signaling pathway. We have previously shown that MmuPV1 E6 interacts with the SMAD2/SMAD3 transcriptional activators to inhibit TGF-β signaling (40). The proteomics data also show a reduction in critical signaling molecules like MAPKs and FGFR (Fig. 8 and 11). Further work is needed to determine the exact mechanism(s) by which MmuPV1 E6 sustains growth under reduced growth factor conditions.

In our studies, we found that MmuPV1 E6 increased the plating efficiency of mouse keratinocytes at lower cell densities (Fig. 2). The enhancement of plating efficiency at lower cell densities likely contributes to the increased proliferation rate of MmuPV1 E6 expressing MKs *in vitro* (Fig. 1A and B). Here, the cells were plated at 0.05 cells/cm^2^, which is a density at which E6 expressing cells demonstrated increased plating efficiency (Fig. 2). A potential mechanism by which MmuPV1 E6 may promote this is by increasing the expression of cell adhesion molecules such as integrins. In our RNAseq analysis, we did not observe a global increase in integrin transcripts but did observe a significant increase in a subset of integrins including Itga2 (6.23-fold increase, *P*-value = 1.93 × 10^−15)^ and Itgb4 (2.35-fold increase, *P*-value = 9.42 × 10^−25^). This specific increase in the expression of Itga2 and Itgb4 might contribute to the increased plating efficiency of MmuPV1 E6 expressing MKs. However, the exact mechanism by which MmuPV1 E6 promotes increased plating efficiency remains elusive, and additional studies are needed to determine the exact mechanism.

Our proteomic and transcriptomic data suggest that MmuPV1 E6 promotes a number of other hallmarks of cancer including evasion of growth suppressors, inhibition of immune response, resistance to cell death, and alterations in DNA damage response (60). Interestingly, the GSEA of the RNAseq data revealed a reduction in a number of growth suppressive pathways in the MmuPV1 E6 expressing MKs including TGF-β signaling and p53 pathways (Fig. 12; Fig. S3). The reduction in expression of genes associated with the p53 pathway is particularly interesting because the high-risk HPV E6 oncoproteins target p53 for degradation to blunt p53 activation (16–18, 21, 73). Our previous AP/MS study of MmuPV1 E6 showed no evidence of MmuPV1 E6 interacting with p53 (40). Therefore, it will be interesting to determine how MmuPV1 E6 inhibits p53 activation and how this might contribute to our *in vitro* phenotypes. With regard to resistance to cell death, the STRING analysis of significantly downregulated proteins (Fig. 8) defined a cluster of proteins related to apoptosis including the reduction of Caspase 8, one of the key proteins for cellular death processes. In addition to this, the GSEA of the RNAseq data also showed a negative enrichment of genes associated with apoptosis (Fig. 11). With regard to inhibiting immune response, the proteomic and transcriptomic data identified E6’s inhibition of genes/proteins important in the innate immune response (including Oas3, Irf3, Irf9, Bst2, and Isg15) and antigen presentation (including Tap1, Tap2, B2m, and MHCI H2-D1) (Fig. 8 to 11). Interestingly, there are some immune-related pathways that were solely altered at the transcriptional level but likely intersect with some of the pathways altered both transcriptionally and at protein steady-state levels, that is, innate immune response and interleukin/MAPK signaling. HPVs are well known to have a number of mechanisms by which they evade the host immune response. Some of these immune evasive activities are shared with HPV oncoproteins E6 and E7 including their ability to inhibit innate immune responses (74–80). In our AP/MS analysis, we found that MmuPV1 E6 interacts with IRF3 which contains an LXXLL motif and could be a potential mechanism by which MmuPV1 E6 alters the innate immune response similar to what has been reported for HPV16 E6 (40, 81). However, we find it interesting that MmuPV1 E6 also targets antigen presentation which is also targeted by the high-risk HPV E5 protein. It is important to note that MmuPV1 does not encode E5 genes, which provides a potential explanation for why MmuPV1 E6 may perform this activity. The genes that MmuPV1 E6 appears to downregulate including Tap1, Tap2, and B2M are transcriptionally activated by the CITA/NLRC5 or CIITA enhancers (82). Through our RNAseq, we find that MmuPV1 E6 does appear to downregulate one of the components of CITA/NLRC5 and CIITA enhancer, RFX5 (1.6-fold decrease, *P*-value = 0.0039). However, further studies are necessary to determine the mechanism by which MmuPV1 E6 targets the innate and adaptive immune responses.

In our proteomic and transcriptomic analysis, we found that MmuPV1 E6 increases the expression of cellular genes, or their protein products associated with DNA damage and DNA repair responses (Fig. 6, 7, 10 and 11). Of these, we see increased levels of proteins associated with replication stress including Fancd2, which has been associated with the expression of high-risk HPV E7 (83). In addition, the cutaneous HPV5 and 8 E6 proteins have also been shown to inhibit the DNA damage response through their interaction with and destabilization of p300, which is thought to contribute to the induction of cutaneous HPV-associated cSCC (84–92). However, MmuPV1 E6 does not interact with p300, and we do not know if and how MmuPV1 E6 may inhibit DNA damage response and DNA repair pathways. Further analysis is needed to understand the underlying mechanisms.

While there are some differences between the RNAseq and proteomic analyses, we find that both analyses are highly congruent with each other. This suggests that most of the changes that we observe in the abundance of proteins are due to changes at the mRNA levels. Based on our analyses, we found that both the RNAseq and Proteomics analyses show an elevation of E2F-responsive genes (Fig. 12 and 13; Table 1). In addition to E2F-responsive genes, we also detected effects on the innate response (a well-known target of high-risk HPV E6 oncoproteins (74, 75, 77, 78, 81, 93)) and antigen presentation.

In summary, we find that MmuPV1 E6 alters cellular processes associated with a number of the hallmarks of cancer (60). We specifically highlighted the ability of MmuPV1 E6 to “sustain proliferative signaling” and to “evade growth suppressors.” Future studies shall be needed to understand more fully the effect of MmuPV1 on other hallmarks of cancer.

## MATERIALS AND METHODS

### Cells

NIH 3T3 murine fibroblasts were obtained from ATCC and grown in Dulbecco’s modified Eagle medium (DMEM) supplemented with 10% calf serum. 293FT cells were obtained from ATCC and grown in DMEM supplemented with 10% fetal bovine serum and pen/strep antibiotic with 200 µg/mL G418. Mouse keratinocytes were isolated from the skin of neonate pups from the FVB/N background as previously described (52, 53). After incubation in phosphate-buffered saline (PBS) containing 10% antibiotics for 2 min, skin pieces were incubated in 0.25% trypsin overnight at 4°C. The epidermis was then separated from the dermis using sterile forceps, minced with a single-edge razor blade, and then stirred for 1 hour at 37°C in F-medium to generate a single-cell suspension. The cells were strained using 0.7 mm membrane (102095-534; VWR) and cultured in F-medium containing 10 mM of the ROCK inhibitor, Y-27632 in the presence of mitomycin C (M4287; Sigma)-treated 3T3 J2 fibroblasts (94). Early passage cells were transduced in keratinocyte serum-free media (1074-001, Gibco) with retroviruses encoding MmuPV1 E6 or vector control (pLXSN). Transduced cells were put under selection at 48 hours following infection with 200 µg/mL G418 (Geneticin, Gibco, 11811-031). Following selection, keratinocytes were cultured in F-medium containing Y-27632 with an appropriate selection agent.

### Plasmids

mE6 was PCR amplified out of the MmuPV genome with Bam/Bgl2 ends and cloned into BamHI cut LXSN to generate a pLXSN-mE6 retroviral expression vector. psPAX2 (Addgene) and pMD2.g (VSVG plasmid, Addgene) plasmids were co-transfected with lentiviral expression vectors to produce lentiviruses for lentiviral transductions. pCL-10A1 (Fisher Scientific) was used for packaging retroviral vectors. pLX304-GFP (Addgene) was used to express GFP in MmuPV1 E6 expressing MKs and pLenti-N (gift from the Munger lab) empty vector was used as empty vector control for lentiviral transductions.

### Retrovirus and lentivirus production

293FT cells were plated into 60 mm tissue culture dishes 24 hours prior to transfection. Plated 293FT cells were changed to DMEM with 10% fetal bovine serum without antibiotics. 293FT cells were transfected with either pLXSN or pLXSN-MmuPV1 E6 and pCL-10A1 packaging vector with Lipofectamine 2000 system (11668-019 Invitrogen). 24 hours post-transfection, 293FT cells were switched to 3 mL of media used for transductions, keratinocyte serum-free media (1074-001, Gibco). 48 hours post-transfection, the supernatant was collected and centrifuged to remove cellular debris and following centrifugation filters through a 0.8-µm filter. Cleared supernatant was used for retroviral transductions. For lentivirus encoding GFP, similar methods were used to generate lentivirus using either pLX304-GFP plasmid or pLenti-N and psPAX2 and pMD2.g packaging plasmids.

### RNA isolation and RT-PCR

Following the selection and establishment of mouse keratinocytes that expressed MmuPV1 E6 or E7, cells were collected and lysed in Trizol. RNA was isolated using a Direct-Zol Miniprep kit (Zymo). cDNA was produced from isolated RNA using QuantiTect Reverse Transcription Kit (205303, Qiagen). Briefly, RNA was treated with DNAse to eliminate cellular DNA. DNAse-treated RNA was then subjected to RT reaction following provided protocol. Following cDNA production, PCR was performed for MmuPV1 E6 using E6-specific primers (Table 1). Following the PCR, products were run on a 2% agarose gel which was stained with ethidium bromide and imaged using Azure Imager 200 (Azure Biosystems).

RNA from RNAseq samples was used to generate cDNA for qRT-PCR analysis of E2F-responsive genes. cDNA was generated as described above. For qRT-PCR, the QuantiTect SYBR Green PCR kit (Qiagen, 204143) was used as per the manufacturer’s protocol. Primers used for qRT-PCR analysis are in Table 2.

**TABLE 2:** Primers used for RT-PCR analyses

Primer	Forward	Reverse
MmuPV1 E6	5′-AGTGCATGGCTGGCAAGAAT-3′	5′-CCAAGTGAAATGGCAAGCCG-3′
MCM7 (mouse)	5′-GAGGCCAGCAGATGTGATATT-3′	5′-GGTGTGAAGCCACGAGATATG-3′
MCM2 (mouse)	5′-CGGAGTATGCGCAAGACTTT-3′	5′-GCCACCAACTGCTTCAGTAT-3′
CCNE2 (mouse)	5′-ATTTGGCTTTGCTGAATGAAGT-3′	5′-CAGTACTCTTTGGTGGTGTCATA-3′
PCNA (mouse)	5’GTTGTCACAAACAAGTAATGTGGAT-3′	5′-CTCAGAAACGTTAGGTGAA-3′
GAPDH (mouse)	5′-GGAGAGTGTTTCCTCGTCCC-3′	5′-ACTGTGCCGTTGAATTTGCC-3′

### Cell counting assays

Confirmed MmuPV1 E6 expressing mouse keratinocyte cell strains were plated on 60 mm dishes with MMC-treated J2 3T3 fibroblast at 1 × 10^5^ cells. 72–96 hours later, MmuPV1 E6 expressing MKs were trypsonized (0.05% trypsin, Gibco, 25300-059) and counted using trypan blue (Gibco, 15250-066) staining on a hemocytometer. Cells were replated and allowed to continue to grow, and counting was repeated every 72–96 hours post-plating for a total of 21 days. Once the total cell number was determined, a fold increase in cell number was calculated at each time point (0, 3, 7, 11, 14, 17, and 21 days) which was then used to determine the total cell number that would accumulate. Growth rate and Doubling time were calculated using the 21-day growth data. Growth rate was calculated by taking ln(Fold Change)/hours and doubling time was determined by ln (2)/Growth Rate. Short-term growth curve was performed by plating 0.25 × 10^5^ cells per well into a six-well plate containing MMC-treated J2 3T3 fibroblasts and each time point was an individual well of the six-well plate. Cells were trypsonized and counted at 1, 2, 3, and 4 days post-plating. The total cell number was calculated at each time point. All these assays were done in F-media without Y-27632

### Plating efficiency assay

Mouse keratinocytes were plated at various doses in a six-well plate including 0.1 × 10^5^, 0.25 × 10^5^, 0.5 × 10^5^, and 1 × 10^5^ cells for each condition. Plates did not contain MMC-treated J2 3T3 fibroblasts. 18 hours post-plating, cells were trypsonized and counted using trypan blue staining. Total cell number was calculated at each dose of cell dose. The assay was done in F-media without Y-27632.

### Competition assay

Mouse keratinocytes co-expressing GFP and MmuPV1 E6 were generated as described above. For GFP and vector control for GFP, mouse keratinocytes were selected using blasticidin selection marker at 7 µg/mL Blasticidin (Gibco, A11139-03). Mouse keratinocytes were maintained under both G410 and blasticidin selection. 1 × 10^5^ cells were plated into 60 mm tissue culture dishes containing MMC-treated J2 3T3 fibroblasts at various ratios of MmuPV1 E6/GFP co-expressing and vector control mouse keratinocytes including 1:1, 1:10, and 1:100. Cells were trypsonized and counted and 1 × 10^5^ cells were plated onto a 60 mm dish containing MMC-treated J2 3T3 fibroblasts for a total of three passages. At each passage, the remaining cells were subjected to flow analysis looking at the percent GFP+ cells at each passage. Cells were treated with DAPI as live dead stains prior to flow cytometry. For flow analysis, an Attune flow cytometer (Thermo Fisher) was used for the collection of Flow data, and FlowJo was used to analyze data. The assay was performed in F-media without Y-27632, and cells were maintained under selection with 200 µg/mL G418 and 7 µg/mL Blasticidin.

### Confluency growth analysis

MmuPV1 E6 or vector control MKs were plated at 1 × 10^5^ cells per well in a six-well plate containing MMC-treated J2 3T3 fibroblasts. A total of six wells were plated per cell line as each well was a time point for this analysis. Cells were trypsonized and counted at 1, 2, 3, 4, 5, and 7 days post-plating. The total cell number was calculated and plotted. Cells were maintained in F-media without Y-27632 and media was changed every 2–3 days.

### Growth factor assay

Prior to seeding MK cell strains, MMC-treated J2 3T3 fibroblasts were plated in F-media without Y-27632 and allowed to adhere overnight. The next day, the F-media was removed and fresh F complete media (F-media containing Insulin, FBS, and EGF) was added to half the wells. In the other half, the media was replaced with F-media without Y-27632, insulin, FBS, and EGF (referred to as F-incomplete media). MmuPV1 E6 expressing and vector control cells were plated at 1 × 10^5^ cells per well. Cells were then trypsonized and counted 48 hours post-plating using trypan blue staining. Fold change was calculated and then plotted.

### Proteomic analysis

Once expression of MmuPV1 E6 was confirmed, frozen cell pellets were subjected to proteomic analysis, specifically quantitative mass spectrometry. Frozen keratinocyte cell pellets were resuspended in lysis buffer (6 M guanidine hydrochloride, 100 mM Tris pH 8) and probe sonicated (Misonix) until homogenized. Proteins were then precipitated by adding methanol to the solution to 90% and centrifuging at 10,000× *g* for 5 minutes. The supernatant was discarded, and the protein pellets were resuspended in digestion buffer (8 M urea, 10 mM TCEP, 40 mM CAA, and 100 mM Tris) and sonicated in a bath sonicator for 5 minutes at 10°C with a program of 10 seconds off/20 seconds on (Qsonica). Endoproteinase Lys-C was added in a 100:1 protein/enzyme ratio, and the samples were incubated at room temperature for 4 hours. The samples were then diluted to 1.5 M urea with 100 mM Tris, and trypsin (Promega) was added in a 50:1 protein/enzyme ratio. After overnight incubation at room temperature, the resulting peptides were acidified to pH 2 with trifluoroacetic acid (TFA), desalted with Strata-X Polymeric solid phase extraction cartridges (Phenomenex), and dried under vacuum.

Each dried peptide sample was resuspended in 0.2% formic acid and loaded onto a 75 µm i.d. × 360 µm o.d. capillary column (New Objective) that was packed in-house with 1.7 µm BEH C18 particles (Waters). Chromatographic separations were performed with a Dionex UltiMate 3000 nano HPLC system (Thermo Scientific). The peptides were loaded in 100% A (0.2% formic acid in water) and eluted with increasing % B (0.2% formic acid in 80% ACN) over a 90-minute gradient. Mass spectrometric detection was performed with an Orbitrap Eclipse (Thermo Scientific), with MS1 scans taken in the Orbitrap (240,000 resolution, 300–1350 m/z scan range, 50 ms maximum injection time, and 1 × 106 AGC target) and MS2 scans in the ion trap (turbo mode, 0.5 m/z isolation width, 150–1350 m/z scan range, 14 ms maximum injection time, and AGC target of 3 × 104).

Mass spectrometry raw files were processed with MaxQuant (version 1.5.2.8) and searched against a database of reviewed mouse proteins plus isoforms (downloaded from UniProt on 12 September 2021). Default parameters were used. Cysteine carbamidomethylation and methionine oxidation were set to fixed and variable modifications, respectively. The “proteinGroups.txt” file was processed by omitting reverse sequences, sequences only identified by site, and contaminants. The data were log2-transformed, and proteins were removed if they were not observed in at least 70% of the samples. The remaining missing values were imputed using Perseus (version 1.6.0.7). Statistical comparisons between groups were performed with two-tailed Student’s *t*-tests. MS raw files were deposited to the MassIVE database with the identifier MSV000089441.

### RNAseq analysis

Following confirmation of expression of MmuPV1 E6, cells were lysed in TRIzol (Thermo Fisher), and total RNA was isolated with the Direct-Zol Miniprep kit (Zymo). RNA quality was assessed with a 4200 Tapestation (Agilent) through the UW-Madison Biotechnology Center. Ribo-depletion, library preparation, and RNA-seq on a NovaSeq 6000 were performed by the Oklahoma Medical Research Foundation Clinical Genomics Center (Oklahoma City, OK). A hybrid index was created by the addition of MmuPV1 and HPV16 E6 sequences to GRCm39. Sequencing reads were aligned to the hybrid index using Docker on HTCondor through the UW-Madison Center for High-Throughput Computing using STAR 2.7.6a (95, 96). Read summarization was performed with featureCounts v1.6.3 and Ensembl annotation release 105. Differential expression analysis was performed with DESeq2 v1.34.0. Gene set enrichment analysis was performed with GSEA v4.3.2 and the hallmark gene set collection from MSigDB (97–99).

### STRING analysis

From the RNAseq and quantitative mass spec analysis, the list of genes/proteins that met the requirement of Log2(FC) >1 or Log2(FC) < −1 and *P*-value < 0.05 was generated. Each list of genes was then separated into upregulated (Log2(FC) >1) or downregulated (Log2(FC) < −1) and uploaded to the STRING database (string-db.org/). Following the uploading of gene sets, STRING interaction networks were generated for each gene/protein list with parameters set to the highest confidence, no text mining, and hidden unconnected nodes. Nodes were pseudo-colored as red for upregulated and blue for downregulated. Images of networks were downloaded for visualization.

### Statistics

All statistical tests were done using MStat software (https://oncology.wisc.edu/mstat/), Graph Pad-Prism (growth curves), or Microsoft Excel.

## Data Availability

The RNA-seq data reported in this paper are available in the BioProject database with the accession number PRJNA1007707.

## SUPPLEMENTAL MATERIAL

Figure S1 mbio.02458-23-s0001.tif Gel images for RT-PCRs for MmuPV E6.

Figure S2 mbio.02458-23-s0002.tif Flow cytometry gating strategy.

Figure S3 mbio.02458-23-s0003.tif GSEA plots for other cellular pathways.

ASM does not own the copyrights to Supplemental Material that may be linked to, or accessed through, an article. The authors have granted ASM a non-exclusive, world-wide license to publish the Supplemental Material files. Please contact the corresponding author directly for reuse.
