# Supplementary figures and images for: MmuPV1 E6 induces cell proliferation and other hallmarks of cancer
Source: mBio. 2023 Oct 31;14(6):e02458-23. doi: 10.1128/mbio.02458-23 (PMC10746199; doi:10.1128/mbio.02458-23)

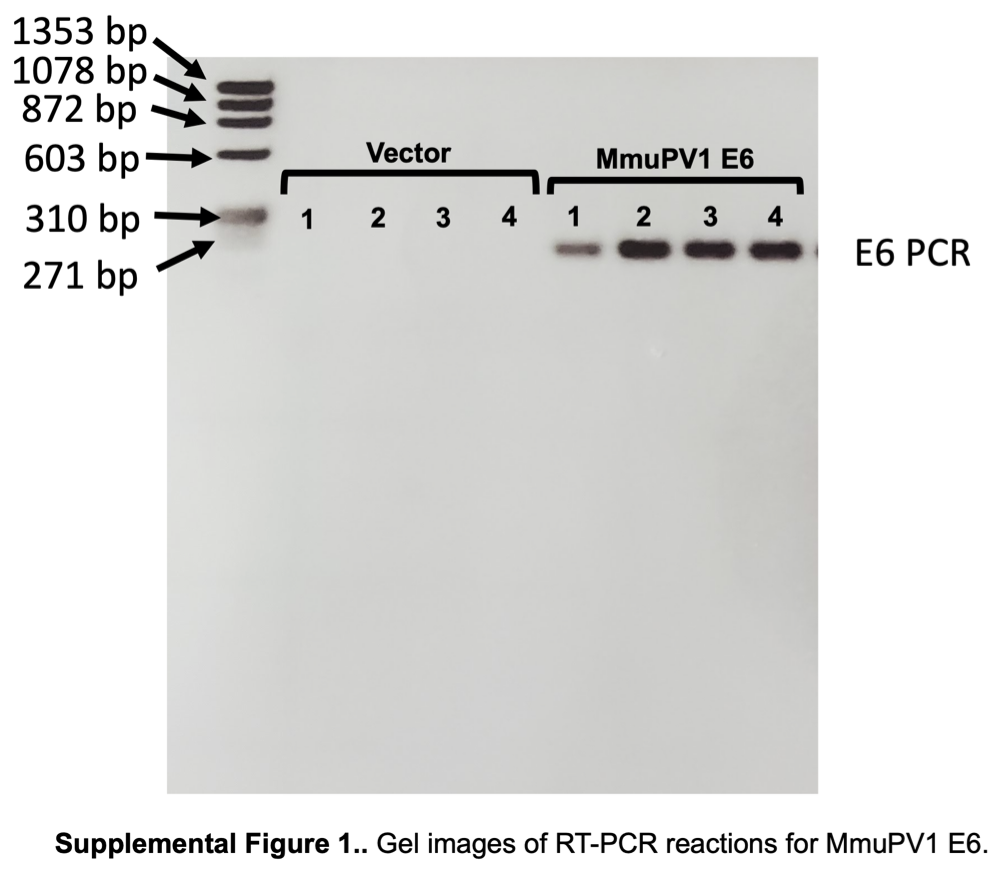

Supplement: Figure S1 — Gel images for RT-PCRs for MmuPV E6. [file mbio.02458-23-s0001.tif]

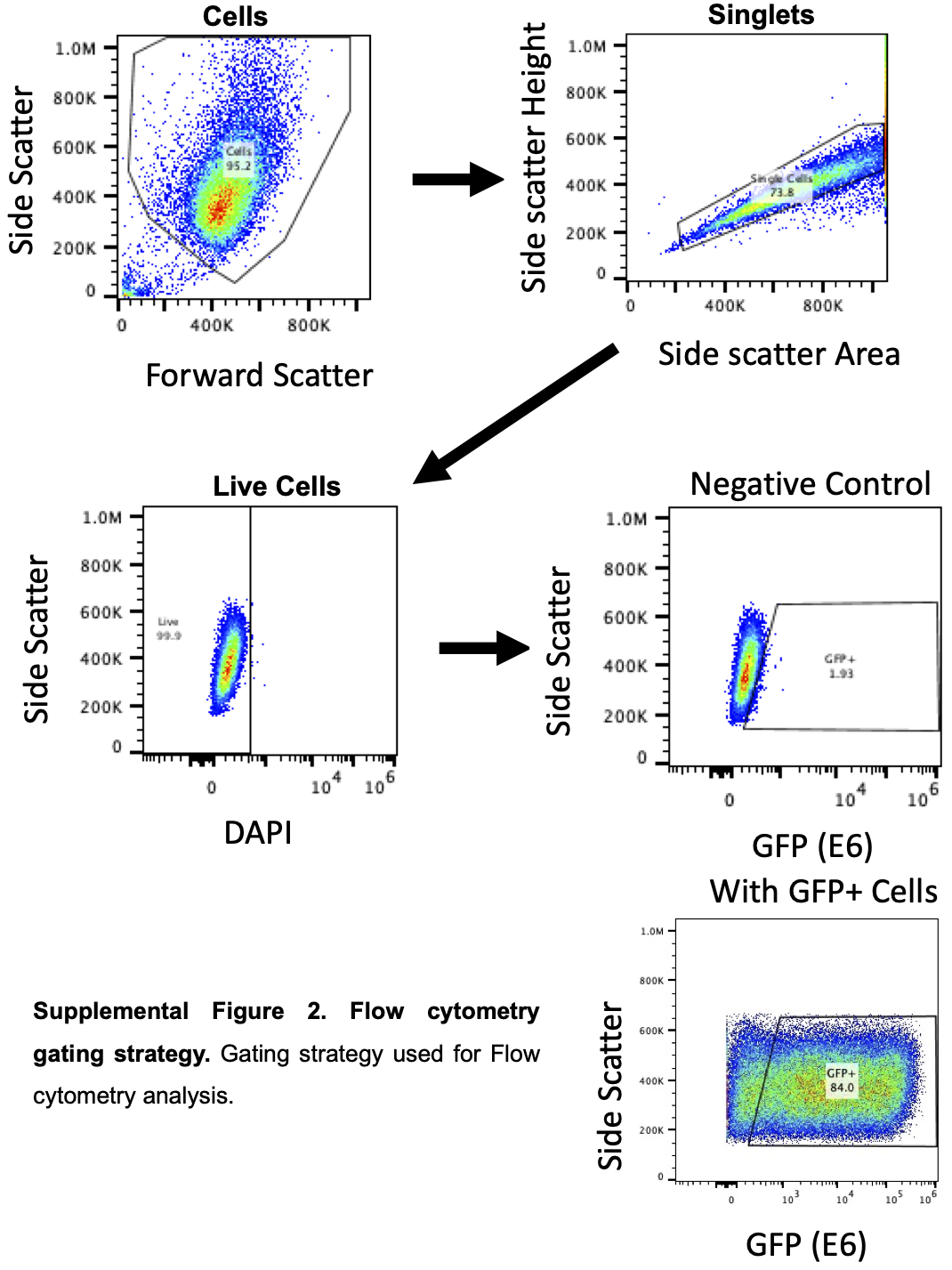

Supplement: Figure S2 — Flow cytometry gating strategy. [file mbio.02458-23-s0002.tif]

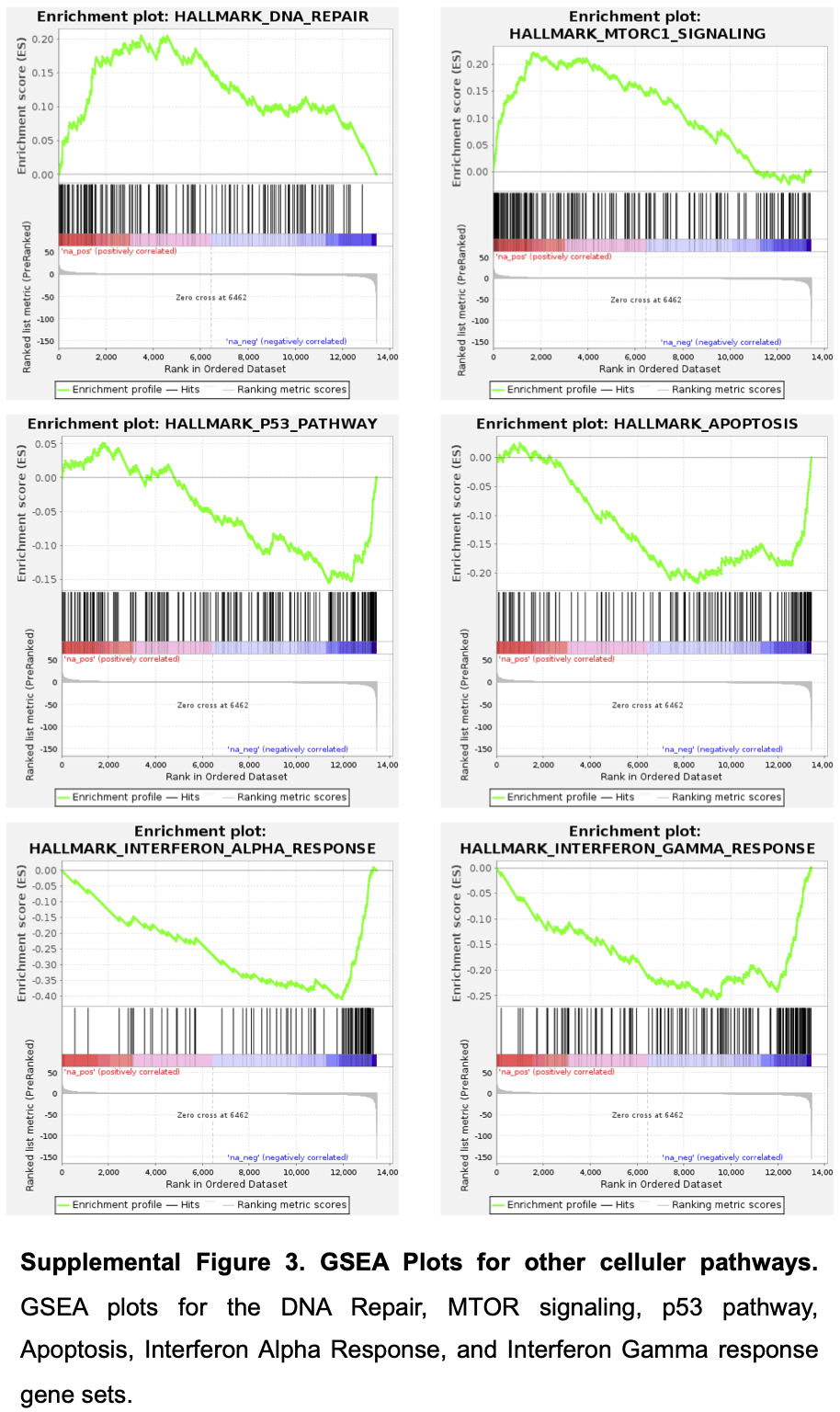

Supplement: Figure S3 — GSEA plots for other cellular pathways. [file mbio.02458-23-s0003.tif]
